# Human Organoids in Infectious Disease Research: From Host–Pathogen Interactions to Vaccine Development

**DOI:** 10.3390/vaccines14100908

**Published:** 2026-10-18

**Authors:** Dorota Zielinska, Marek Fol, Magdalena Druszczynska

**Affiliations:** Department of Immunology and Infectious Biology, Faculty of Biology and Environmental Protection, University of Lodz, Banacha 12/16, 90-237 Lodz, Poland; dorota.zielinska@edu.uni.lodz.pl (D.Z.); marek.fol@biol.uni.lodz.pl (M.F.)

**Keywords:** organoids, vaccines, host–pathogen interactions, infectious disease modeling, immune response, preclinical models, precision vaccinology

## Abstract

**Background/Objectives:** Human organoids have emerged as physiologically relevant models bridging conventional two-dimensional cultures and animal models. This review summarizes advances in their application to infectious disease modeling and vaccine research, with emphasis on host–pathogen interactions, immune responses, and translational potential. **Methods**: A narrative review was conducted using targeted searches of PubMed, Scopus, Web of Science Core Collection, and ScienceDirect. Publications from January 2000 to June 2026 were considered, and relevant evidence was qualitatively synthesized according to established applications, proof-of-concept studies, and prospective uses. **Results:** Human organoids have advanced the study of viral, bacterial, parasitic, and fungal infections by enabling investigation of pathogen entry, tissue tropism, barrier integrity, and immune responses in human-relevant systems. Immune and tonsil-derived organoids have also demonstrated applications in evaluating vaccine-induced immunity, comparing vaccine formulations, investigating mechanisms of immunogenicity, and identifying potential correlates of protection. Integration with single-cell and spatial omics, advanced imaging, and computational approaches is further expanding their utility. However, incomplete immune and vascular complexity, inter-model variability, limited standardization, scalability constraints, and insufficient validation against in vivo and clinical outcomes remain important challenges. **Conclusions:** Human organoids represent complementary human-relevant platforms with substantial potential for mechanistic and translational vaccinology. Further standardization and prospective validation are required to establish their predictive value for vaccine development.

## 1. Introduction

Vaccination remains one of the most successful public health interventions, preventing millions of deaths annually and substantially reducing the burden of infectious diseases worldwide. Despite remarkable advances in vaccine technologies, including mRNA-based platforms, viral vectors, recombinant protein subunits, and nanoparticle formulations, the overall development pipeline remains lengthy, costly, and associated with high attrition rates, particularly during late-stage preclinical and clinical evaluation. A major limiting factor in vaccine development is the insufficient predictive value of current preclinical models for human immune responses.

Although animal models remain indispensable for studying systemic immunity, disease pathogenesis, and vaccine safety, their ability to predict human immune responses is often limited by interspecies differences in immune system organization, receptor expression, cytokine signaling, tissue physiology, and pathogen tropism. Consequently, findings obtained in animal models do not always translate into human clinical outcomes, particularly for pathogens exhibiting marked host specificity [1,2,3,4,5]. Similarly, conventional two-dimensional (2D) cell culture systems, although inexpensive and experimentally convenient, fail to reproduce the three-dimensional architecture, cellular heterogeneity, epithelial polarity, extracellular matrix interactions, and tissue-specific microenvironment that govern host–pathogen interactions in vivo. As most 2D cultures consist of transformed or immortalized cell lines representing a single cell type, they frequently provide an oversimplified model of human tissue physiology and immune responses [6,7,8]. In recent years, human organoid technology has emerged as a transformative approach in biomedical research. Organoids are three-dimensional structures derived from pluripotent or adult stem cells that self-organize into tissue-specific architectures, recapitulating many structural, cellular, and functional characteristics of native organs, including epithelial polarity, multicellular composition, and tissue-specific differentiation programs [9,10,11]. In addition to organoids generated from healthy tissues, patient-derived organoids largely preserve the genetic, molecular, and phenotypic characteristics of the donor tissue, making them valuable models for studying inter-individual variability, disease pathogenesis, and therapeutic responses [9,12,13].

Recent advances in organoid technology have made it possible to incorporate stromal, vascular, neuronal, and immune components into tissue-specific models [6,7,8]. This has broadened their use in infection research, where the interactions between pathogens, epithelial barriers, and local immune responses often depend on the tissue environment. Unlike conventional cell cultures, organoids retain aspects of native tissue architecture and cellular diversity, allowing researchers to investigate pathogen entry and replication, tissue tropism, epithelial damage, and host responses in a human-derived system. Such models have been used to study a range of viral, bacterial, parasitic, and fungal infections [6,7,8,14]. Immune-enhanced and patient-derived organoids, as well as organ-on-chip systems, have also opened opportunities to investigate questions relevant to translational immunology and vaccine research [9,13,15,16]. This review examines the use of human organoids in infectious disease research and their relevance to vaccine development. In addition to studies of host–pathogen interactions, it discusses the application of organoid models to antigen discovery, the assessment of vaccine-induced immune responses, and the evaluation of vaccine candidates. Attention is given to the strength of the available evidence, distinguishing experimentally demonstrated applications from proof-of-concept studies and potential future uses. The review also considers the extent to which findings obtained in organoid models correspond to results from in vivo studies and human vaccination datasets, as well as the limitations affecting their translational relevance. Finally, it discusses outstanding methodological challenges and future research directions, including the need for standardized protocols and further validation, alongside emerging approaches such as immune-enhanced organoids, organ-on-chip systems, multi-omics, and personalized vaccinology.

## 2. Materials and Methods

This narrative review synthesizes current knowledge on the application of human organoid systems in infectious disease research and vaccine development. The literature search focused on studies addressing host–pathogen interactions, tissue-specific immune responses, infectious disease modelling, and applications of organoid systems in vaccine research. Relevant literature was identified through targeted searches of PubMed, Scopus, Web of Science Core Collection, and ScienceDirect, covering publications from January 2000 to June 2026. The search strategy combined terms related to organoid biology, infectious diseases, immunology, and vaccinology. Search concepts included, but were not limited to, organoid, human organoid, intestinal organoid, lung organoid, brain organoid, liver organoid, infectious disease, host–pathogen interaction, viral infection, bacterial infection, parasitic infection, fungal infection, immune response, mucosal immunity, vaccine, vaccine development, antigen discovery, and immunogenicity. Boolean operators (AND, OR) were used to combine the search concepts. Google Scholar was used as a supplementary source to identify additional relevant publications and citation links. PubMed Central was not treated as an independent bibliographic database and was used primarily to access full-text articles when required. Publications were considered eligible when they addressed human organoid-based infectious disease research, host–pathogen interactions, immune responses, or vaccine-related applications. Original research articles, experimental and translational studies, and relevant reviews were considered. Conference abstracts, editorials, letters without relevant primary data, duplicate publications, non-English publications, and studies outside the scope of human organoid-based infectious disease or vaccine research were excluded. Study selection was based on relevance to the objectives and scope of the review, with particular attention to the organoid model, infectious agent or vaccine platform, experimental design, biological or immunological endpoints, and translational relevance. Owing to the broad scope and substantial heterogeneity of organoid models, tissue types, pathogens, vaccine platforms, and experimental outcomes, the findings were synthesized qualitatively rather than quantitatively. To provide a more accurate representation of the current evidence, applications were further considered according to their level of evidence as established applications, proof-of-concept studies, or prospective/potential applications.

## 3. Human Organoid Characteristics

**Figure 1:**
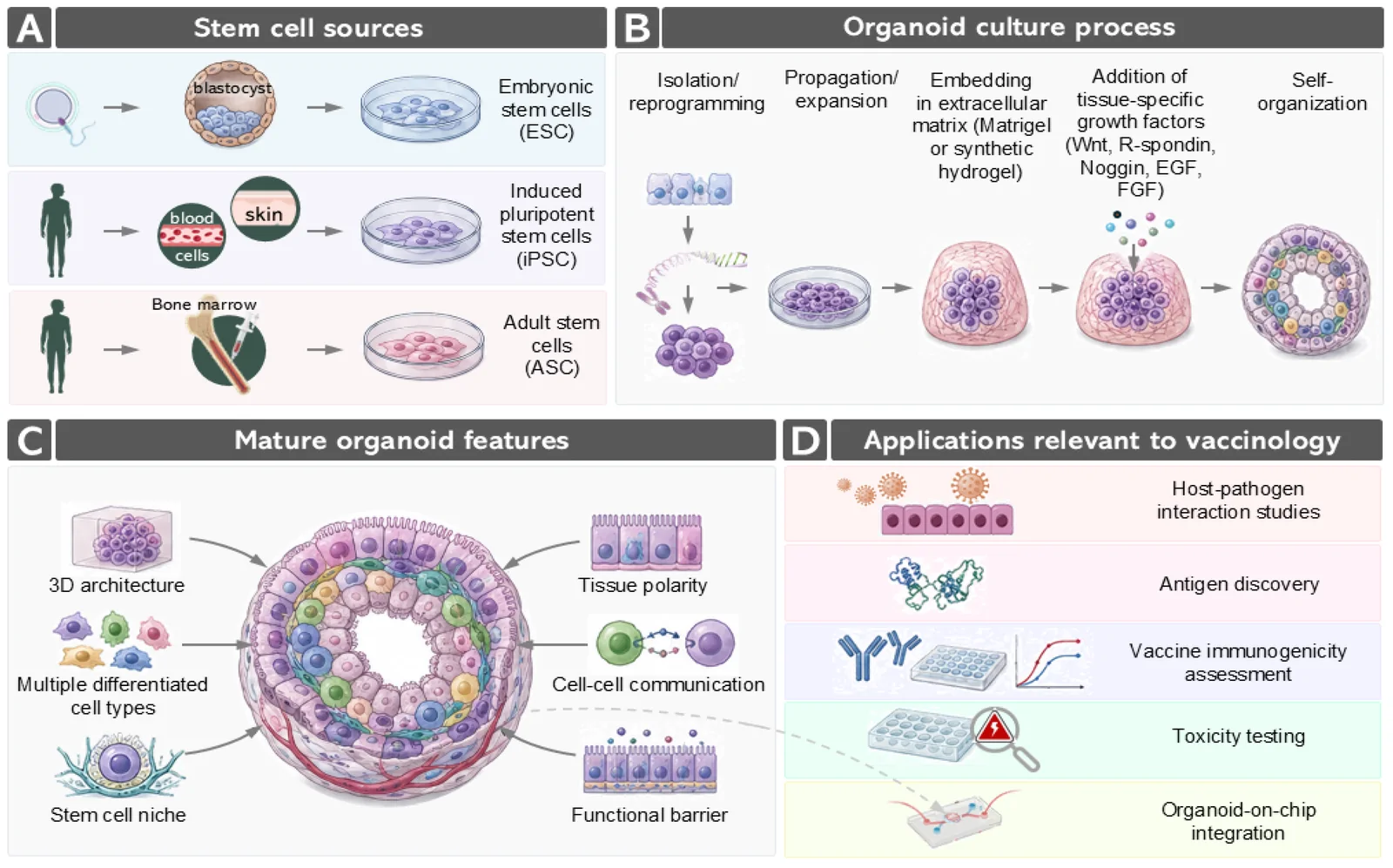
Generation and biological characteristics of human organoids. Organoids are generated from embryonic stem cells (ESCs), induced pluripotent stem cells (iPSCs), or adult stem cells (ASCs) through sequential expansion, extracellular matrix embedding, and tissue-specific differentiation, resulting in self-organizing three-dimensional structures that recapitulate key structural and functional features of native tissues. Their physiological relevance makes organoids a versatile platform for investigating host–pathogen interactions, identifying and validating vaccine antigens, evaluating vaccine immunogenicity and safety, assessing toxicity, and advancing next-generation preclinical models through integration with organ-on-chip technologies. Abbreviations: Wnt—large family of secreted protein growth factors that have been identified in animals from hydra to humans (in humans 19 Wnt proteins have been identified), and encoded by highly conserved Wnt genes; R-spondin—protein family, which consists of four evolutionarily conserved secreted proteins RSPO1-4 (best characterized as growth factors for adult stem cells); Noggin—32 kDa glycoprotein, originally isolated from the aquatic-frog genus Xenopus, which supports maintenance of human embryonic stem cells in vitro; EGF (Epidermal growth factor)—53 amino acid polypeptide, which is involved in the regulation of cell proliferation; FGF (Fibroblast growth factors)—large family of cell-signaling proteins that regulate a broad spectrum of biological functions, including cellular proliferation, survival, migration, and differentiation based on [19,20,21,22,23,24].

Human organoids are three-dimensional (3D), self-organizing multicellular structures generated from pluripotent stem cells (PSCs), including embryonic stem cells (ESCs) and induced pluripotent stem cells (iPSCs), or from tissue-resident adult stem cells (ASCs). Under appropriate culture conditions that recapitulate key developmental signaling pathways, these stem cells undergo coordinated proliferation, lineage specification, and spatial organization, giving rise to miniature tissue-like structures that reproduce many of the architectural, molecular, and functional characteristics of their in vivo counterparts (Figure 1) [10,17,18]. Unlike conventional two-dimensional (2D) cell cultures, organoids preserve essential features of tissue complexity, including cellular heterogeneity, epithelial polarity, stem cell niches, and dynamic cell–cell as well as cell–extracellular matrix interactions. These properties allow organoids to recreate physiological processes that are difficult or impossible to model using monolayer cultures, such as tissue morphogenesis, differentiation gradients, barrier formation, and organ-specific responses to pathogens or therapeutic interventions. The distinction between organoids and conventional co-culture systems lies not simply in the number of cell types present, but also in how cellular composition and tissue organization are established. Conventional co-culture systems typically combine selected cell populations to investigate defined cellular interactions, whereas organoids develop through coordinated stem-cell proliferation and differentiation, enabling these interactions to be studied within a self-organized, tissue-like architecture. Nevertheless, co-culture systems offer greater experimental control over selected cellular components and remain valuable for investigating specific mechanisms. Moreover, organoids can be combined with additional cell types to incorporate immune, stromal, or endothelial components. These approaches are therefore complementary rather than interchangeable, and their suitability depends on the biological question being investigated [6,7,9].

Organoid formation relies on the intrinsic capacity of adult or pluripotent stem cells to undergo self-renewal and self-organization when cultured within a supportive extracellular matrix and exposed to tissue-specific developmental signals. Most organoid cultures are embedded in matrix scaffolds such as Matrigel or synthetic hydrogels, which provide structural support while permitting cell migration and tissue remodeling [25]. Culture media are supplemented with combinations of growth factors and morphogens, including Wingless/Integrated (Wnt) ligands, R-spondin, Noggin, epidermal growth factor (EGF), fibroblast growth factors (FGFs), and inhibitors of bone morphogenetic protein (BMP) signaling to recapitulate developmental pathways regulating stem cell maintenance and organogenesis (Figure 1) [17,26]. Although the core principles of organoid culture are shared across systems, the composition of signaling molecules is tailored to the tissue of origin, reflecting the distinct molecular requirements governing the development and homeostasis of intestinal, pulmonary, hepatic, cerebral, and other organ-specific tissues [9,10].

A defining characteristic of organoids is their ability to generate diverse, tissue-specific cell populations that self-organize into structures closely resembling native organs. Mature organoids comprise multiple differentiated cell types arranged in physiologically relevant spatial patterns [9,10]. For example, intestinal organoids include absorptive enterocytes, goblet cells, enteroendocrine cells, Paneth cells, and Lgr5^+^ stem cells organized into crypt-like and villus-like domains, whereas airway organoids reproduce basal, club, goblet, and ciliated epithelial cells [26,27]. Likewise, cerebral organoids generate region-specific neuronal and glial populations that recapitulate key aspects of early human brain development [18]. This multicellular composition enables the investigation of complex biological interactions between different cell types during infection, inflammation, tissue repair, and immune activation [6,7]. Importantly, organoids retain many genetic, transcriptomic, proteomic, and epigenetic characteristics of the donor tissue [9]. Adult stem cell-derived organoids preserve patient-specific genetic alterations and disease-associated phenotypes during long-term expansion, making them particularly valuable for precision medicine and individualized therapeutic testing [12,13,28]. In contrast, PSC-derived organoids recapitulate early stages of human organogenesis and provide unique opportunities to investigate embryonic development, congenital disorders, and tissue maturation processes that cannot be directly examined in humans [11,17].

The physiological relevance of organoids has led to their widespread adoption in infectious disease research [6,7]. Organoid models faithfully reproduce tissue-specific receptor expression, pathogen tropism, epithelial barrier function, and innate immune responses, enabling detailed investigation of host–pathogen interactions under physiologically relevant conditions [6,8]. During the COVID-19 pandemic, respiratory and intestinal organoids became indispensable tools for studying SARS-CoV-2 entry, viral replication, host responses, and antiviral drug efficacy [29]. Similar approaches have since been applied to influenza viruses, respiratory syncytial virus, rotavirus, norovirus, hepatitis viruses, and emerging zoonotic pathogens, establishing organoids as versatile systems for translational vaccinology [8,30]. Nevertheless, despite these significant advantages, current organoid models remain simplified representations of native human organs. Most conventional organoids lack vascularization, lymphatic drainage, neuronal innervation, and fully functional immune compartments, limiting their ability to reproduce complex systemic responses [9,31]. Furthermore, culture-to-culture variability, incomplete tissue maturation, and dependence on animal-derived extracellular matrices continue to limit reproducibility and standardization across laboratories [25,32]. Ongoing advances in bioengineering, including synthetic extracellular matrices, microfluidic organoid-on-chip systems, immune cell co-cultures, and vascularized organoids, are expected to overcome many of these limitations and further increase the translational value of organoid technology for infectious disease research and vaccine development [15,32,33].

### 3.1. Organoid Platforms for Modeling Infectious Diseases

The expanding repertoire of human organoid models has created unprecedented opportunities for investigating host–pathogen interactions across virtually every organ system susceptible to infectious diseases [6,7]. Since pathogens exhibit pronounced tissue tropism, selecting an organoid that faithfully reproduces the primary site of infection is essential for accurately modeling disease pathogenesis and evaluating vaccine-induced protection [6,9]. Consequently, organoids derived from different organs have become complementary platforms rather than interchangeable experimental systems. Depending on their tissue of origin, organoids differ considerably in cellular composition, structural organization, receptor expression, innate immune competence, and physiological functions [10]. These differences directly determine their suitability for studying specific pathogens and vaccine platforms (Table 1). Respiratory organoids are particularly valuable for investigating airborne viruses, intestinal organoids for enteric pathogens and oral vaccines, liver organoids for hepatotropic infections, and brain organoids for neurotropic viruses [6,9]. More recently, lymphoid and immune organoids have emerged as sophisticated systems for studying adaptive immune responses, germinal center formation, antigen presentation, and antibody maturation, thereby extending organoid applications beyond disease modeling into functional vaccinology [33]. Importantly, organoid selection should not merely reflect the anatomical site of infection but also the biological question being addressed. While epithelial organoids are highly suitable for studying pathogen entry, replication, and epithelial innate immunity, immune organoids are better suited for investigating vaccine immunogenicity and adaptive immune activation. Increasingly, integrated multi-organoid systems combine different tissue types to better mimic the systemic nature of infectious diseases and vaccination responses [32,34].

#### 3.1.1. Respiratory Organoids

Respiratory organoids represent one of the most extensively used organoid models in infectious disease research, particularly following the COVID-19 pandemic [6,9]. These models reproduce the complex cellular architecture of the human airway epithelium, including basal cells, club cells, goblet cells, ciliated epithelial cells, and alveolar type II pneumocytes [27]. Importantly, these organoids express physiologically relevant levels of viral entry factors, including angiotensin-converting enzyme 2 (ACE2) and transmembrane serine protease 2 (TMPRSS2), enabling faithful modeling of respiratory viral infections [29,30]. Respiratory organoids have been widely applied to investigate influenza viruses, SARS-CoV-2, respiratory syncytial virus (RSV), adenoviruses, and emerging zoonotic respiratory pathogens [7]. Beyond characterizing viral attachment, entry, and replication, these models enable detailed analysis of epithelial barrier integrity, interferon signaling, inflammatory cytokine production, tissue regeneration, and host antiviral responses [40]. Owing to their human-specific physiology, respiratory organoids also represent valuable platforms for evaluating mucosal immune responses, antiviral compounds, and vaccine-induced protection against respiratory pathogens [9,33].

#### 3.1.2. Intestinal Organoids

The intestinal epithelium constitutes one of the largest immune interfaces in the human body and serves as the primary portal of entry for numerous bacterial, viral, and parasitic pathogens [41,42]. Human intestinal organoids reproduce crypt-villus organization and contain multiple differentiated epithelial cell populations, including enterocytes, Paneth cells, goblet cells, enteroendocrine cells, tuft cells, and intestinal stem cells [10,26]. A characteristic feature of three-dimensional intestinal organoids is the formation of a central lumen surrounded by a polarized epithelial layer, with the apical surface facing the lumen and the basolateral surface oriented toward the surrounding extracellular matrix. This organization recapitulates key aspects of intestinal tissue architecture and enables investigation of epithelial barrier function, secretion, and interactions between luminal pathogens and host cells [26]. These models have revolutionized research on numerous enteric viral, bacterial and parasitic pathogens including human norovirus, rotavirus, *Salmonella enterica*, pathogenic *Escherichia coli*, *Shigella* spp. or *Cryptosporidium parvum* [6,7,35,43]. Within vaccine research, intestinal organoids provide physiologically relevant platforms for evaluating oral vaccine candidates, intestinal barrier integrity, antigen uptake, epithelial innate immunity, and microbiota-dependent modulation of vaccine responses [33]. Their ability to preserve donor-specific genetic backgrounds further supports investigations into inter-individual variability in mucosal vaccine efficacy [13,28].

#### 3.1.3. Liver Organoids

Liver organoids closely reproduce hepatic architecture and contain hepatocyte-like cells together with cholangiocyte populations responsible for biliary function [36,44]. These systems have been successfully applied to investigate infections caused by hepatitis B virus (HBV), hepatitis C virus (HCV), and hepatitis E virus (HEV), enabling detailed analysis of viral replication, host responses, and tissue injury in a human-relevant environment [44,45,46]. Beyond infection biology, liver organoids are increasingly explored for preclinical assessment of hepatic safety, inflammatory responses, and metabolic effects associated with vaccine candidates and novel delivery systems. Furthermore, they provide promising platforms for investigating liver-specific innate immune pathways involved in antiviral defense and systemic immune activation [33,44].

#### 3.1.4. Brain Organoids

Human brain organoids have transformed the study of neurotropic pathogens by providing access to early stages of human neurodevelopment that cannot be directly investigated in vivo [9,18]. They contain diverse neuronal and glial populations organized into region-specific structures resembling the developing cerebral cortex. Brain organoids gained worldwide attention during the Zika virus epidemic, when they demonstrated that Zika virus selectively infects neural progenitor cells, leading to impaired neurogenesis and microcephaly-like phenotypes [38,47,48]. Subsequently, they have been applied to investigate the neuropathogenesis of several clinically important viruses, including Japanese encephalitis virus, West Nile virus, herpes simplex virus, cytomegalovirus, rabies virus, and SARS-CoV-2-associated neurological manifestations [9,18,47,49]. Although their application in vaccinology remains at an early stage, brain organoids are increasingly recognized as promising platforms for evaluating neurovirulence, neurotoxicity, and central nervous system responses during the preclinical assessment of live-attenuated vaccines and viral vector-based vaccine platforms.

#### 3.1.5. Lymphoid and Immune Organoids

Among the most recent advances in organoid technology are lymphoid and engineered immune organoids, which extend conventional epithelial models by recapitulating key features of secondary lymphoid tissues, including lymph nodes, tonsils, and germinal centers [9,33]. These models are designed to reproduce the specific microenvironments in which adaptive immune responses are initiated and maintained. Recent bioengineering approaches have enabled the incorporation of dendritic cells, macrophages, T and B lymphocytes, stromal cells, and extracellular matrix components into three-dimensional immune cultures that support antigen presentation, lymphocyte activation, germinal center formation, affinity maturation, class-switch recombination, and memory B-cell differentiation [16,33]. Owing to their ability to model human adaptive immunity in a physiologically relevant setting, immune organoids are emerging as promising platforms for preclinical evaluation of vaccine immunogenicity. They enable functional assessment of antigen-specific immune responses, comparison of vaccine formulations and adjuvants, investigation of correlates of protection, and may facilitate prediction of inter-individual variability in vaccine responsiveness [16,39].

#### 3.1.6. Organoid Models of Infection- and Inflammation-Associated Carcinogenesis

Organoids are also being used to investigate how infection and chronic inflammation may contribute to cancer development. Gastric organoid models have helped researchers examine interactions between *Helicobacter pylori* and the gastric epithelium, including bacterial colonization, inflammatory signaling, and epithelial damage and repair, all of which may be relevant to the early stages of gastric carcinogenesis [50]. A more direct link between bacterial exposure and cancer-associated changes was demonstrated by Pleguezuelos-Manzano et al., who exposed human intestinal organoids to genotoxic pks-positive *Escherichia coli* over several months. The treatment produced a distinct mutational signature resembling patterns subsequently identified in colorectal cancer [51]. Hibiya et al. approached the question from a different perspective, using murine colonic organoids to examine the effects of prolonged inflammatory stimulation. They observed persistent changes in epithelial properties, providing a model for studying processes involved in inflammation-associated colorectal carcinogenesis [52]. Although these studies offer useful insight into the early cellular and molecular changes associated with tumor development, organoid models cannot yet reproduce the full course of human carcinogenesis or the complex interactions between epithelial, immune, and stromal cells in vivo.

#### 3.1.7. Emerging Multi-Organoid and Organoid-on-Chip Platforms

Although individual organoids faithfully reproduce tissue-specific biology, infectious diseases frequently involve coordinated interactions among multiple organs and immune compartments [9,33]. Consequently, increasing attention has focused on interconnected organoid systems integrated with microfluidic technologies that more closely recapitulate systemic human physiology [15,53]. Organoid-on-chip platforms combine dynamic perfusion with multiple tissue compartments, enabling simulation of tissue–tissue communication, immune cell trafficking, cytokine exchange, pharmacokinetics, and systemic inflammatory responses [15,32]. These integrated systems are expected to improve the physiological relevance of preclinical vaccine evaluation by reproducing complex biological interactions that cannot be modeled using isolated organoids alone [32,34]. In the future, multi-organ platforms integrating respiratory, intestinal, hepatic, vascular, and lymphoid organoids may provide human-relevant systems for simultaneous assessment of vaccine efficacy, safety, biodistribution, and tissue-specific adverse responses, thereby improving the predictive value of preclinical vaccine testing [15,34].

## 4. Human Organoids as Models of Infectious Diseases

The outcome of infectious diseases is determined by a dynamic interplay between invading pathogens and host tissues. Successful colonization, replication, dissemination, and immune evasion depend not only on pathogen virulence factors but also on the structural and immunological properties of the infected organ. Consequently, understanding host–pathogen interactions is fundamental for rational vaccine design, as protective immunity must target the earliest stages of infection before irreversible tissue damage or systemic spread occurs. Human organoids have emerged as powerful experimental systems capable of reproducing these early events with a degree of physiological relevance that cannot be achieved using conventional 2D cell cultures. By maintaining tissue-specific architecture, cellular heterogeneity, and functional epithelial barriers, organoids provide unprecedented opportunities to investigate the molecular mechanisms governing pathogen invasion and host defense while simultaneously identifying correlates of vaccine-induced protection [7,9,10,11].

### 4.1. Viral Infections

#### 4.1.1. Respiratory Viruses

Respiratory viruses remain among the leading causes of global morbidity and mortality, accounting for millions of hospitalizations and hundreds of thousands of deaths annually [54]. Seasonal influenza viruses, respiratory syncytial virus (RSV), and severe acute respiratory syndrome coronavirus 2 (SARS-CoV-2) continue to pose major public health challenges, emphasizing the need for predictive human-relevant preclinical models to support vaccine development [55,56,57]. Historically, studies of respiratory virus biology have relied on immortalized cell lines, primary airway cultures, and animal models. Although these systems have greatly advanced our understanding of viral pathogenesis, they frequently fail to reproduce the structural complexity, cellular heterogeneity, and human-specific immune responses of the respiratory tract, limiting their translational value [7,9]. Recent advances in respiratory organoid and ex vivo airway culture systems have highlighted these limitations by demonstrating important differences in viral entry, replication, and host responses compared with conventional models [29,30,57,58]. Human respiratory organoids closely recapitulate the architecture of the airway epithelium, containing basal, ciliated, goblet, club, and alveolar epithelial cells organized into polarized three-dimensional structures. They preserve physiologically relevant expression of viral entry receptors, epithelial barrier function, mucus production, ciliary activity, and innate immune signaling, providing a robust platform for investigating the earliest stages of respiratory viral infection and host defence [27,29,57]. Beyond modeling viral pathogenesis, respiratory organoids have become valuable tools for vaccine research, enabling evaluation of viral tropism, epithelial and innate immune responses, neutralizing antibody activity, and tissue repair. Patient-derived organoids further support investigations of host-specific susceptibility, chronic respiratory diseases, and inter-individual variability in vaccine responsiveness, highlighting their potential for precision vaccinology [13,59]. Future integration with immune cell co-cultures, vascularized organoids, organ-on-chip technologies, and artificial intelligence is expected to further improve the predictive power of these models for the development of next-generation respiratory vaccines [60,61].

##### SARS-CoV-2

The COVID-19 pandemic represented a major turning point in organoid research. Within months of the emergence of SARS-CoV-2, multiple laboratories established airway, bronchial, alveolar, nasal, and lung organoid models that rapidly became indispensable tools for investigating viral biology (Table 2) [30,62,63,64,65]. These systems demonstrated that SARS-CoV-2 exhibits pronounced cellular tropism toward ciliated epithelial cells and alveolar type II pneumocytes expressing ACE2 and TMPRSS2, confirming observations from primary human tissues while enabling mechanistic dissection of infection pathways [29,30,66]. Respiratory organoids revealed several previously unrecognized aspects of SARS-CoV-2 pathogenesis. Single-cell transcriptomic analyses demonstrated marked heterogeneity in receptor expression across epithelial populations, explaining differential susceptibility within the respiratory tract. In addition, infected organoids showed delayed and attenuated type I and III interferon responses relative to influenza virus infection, consistent with the ability of SARS-CoV-2 to evade early innate antiviral signaling [29,30,67]. These findings provided mechanistic insights into early asymptomatic transmission and identified innate immune pathways that may represent potential targets for antiviral therapies and the rational design of future vaccine adjuvants [29]. Respiratory organoids have also emerged as promising platforms for evaluating neutralizing antibody responses induced by vaccination or natural infection. In experimental settings, organoids pre-incubated with immune sera or monoclonal antibodies before viral challenge enable assessment of viral entry inhibition and viral replication in a physiologically relevant epithelial environment. Compared with conventional immortalized cell lines, these models better preserve tissue architecture, cellular heterogeneity, and physiological receptor expression, thereby providing a more physiologically relevant platform for evaluating neutralizing activity [29,30,68]. Furthermore, organoid systems have enabled comparative evaluation of antibody efficacy against emerging SARS-CoV-2 variants, supporting rapid assessment of immune escape and informing vaccine update strategies [29,64]. Beyond humoral immunity, emerging immune-enhanced respiratory organoid models incorporating macrophages, dendritic cells, and lymphocytes have begun to facilitate investigations of local antiviral immune responses, epithelial–immune cell interactions, and mechanisms of host defence. Although these systems remain at an early stage of development, they represent promising platforms for studying mucosal immunity and, potentially, vaccine-induced immune responses [33,69]. Although still technically challenging, these immune-enhanced respiratory organoids increasingly resemble the airway mucosal immune environment encountered during natural infection and vaccination. Patient-derived respiratory organoids have enabled investigation of host-specific determinants of SARS-CoV-2 susceptibility, including aging, chronic pulmonary diseases, smoking-associated epithelial dysfunction, and interindividual variability in antiviral responses, thereby expanding their utility for precision vaccinology and personalized therapeutic development [59,70]. In parallel, respiratory organoids have been increasingly applied to compare the infectivity, tissue tropism, and immune evasion characteristics of emerging SARS-CoV-2 variants. These studies demonstrated variant-specific differences in cellular tropism, replication efficiency, and sensitivity to neutralizing antibodies, highlighting the value of organoid models for rapid assessment of newly emerging variants and supporting timely vaccine updates [64,70].

##### Influenza Virus

Influenza viruses continue to pose a substantial public health challenge due to continuous antigenic drift and the periodic emergence of pandemic strains. Effective influenza vaccine development therefore requires experimental systems capable of rapidly evaluating newly emerging variants while accurately predicting human susceptibility. Human-adapted influenza viruses preferentially bind to α2,6-linked sialic acid receptors, which are predominantly expressed in the human upper respiratory tract, enabling efficient viral attachment, entry, and replication [71]. Differentiated airway epithelial organoids and primary airway epithelial cultures preserve key features of the native respiratory epithelium, including ciliated, secretory, and basal cell populations, which are critical determinants of viral tropism and host susceptibility [27,79]. Studies using human airway epithelial and air–liquid interface (ALI) systems have demonstrated that influenza viruses preferentially infect ciliated epithelial cells, resulting in epithelial injury, loss of cilia, and disruption of mucociliary clearance, which are central features of influenza pathogenesis [72,80,81]. These structural changes are accompanied by robust activation of innate immune responses, which contribute to antiviral defence and disease severity [73]. Organoid-based and primary airway epithelial models have also been used to investigate replication kinetics and cell-type specificity of seasonal, avian, and pandemic influenza strains. These systems reproduce key aspects of interspecies transmission and host adaptation, thereby providing relevant experimental platforms for evaluating viral fitness and informing vaccine strain selection strategies [81].

##### Respiratory Syncytial Virus

Respiratory syncytial virus (RSV) remains a leading cause of lower respiratory tract infections in infants and a major contributor to severe disease in elderly and immunocompromised individuals [82]. Despite decades of research, vaccine development has been hindered by the lack of physiologically relevant human models that accurately recapitulate RSV infection of the airway epithelium [83]. The development of differentiated human airway epithelial models, including ALI cultures and, more recently, airway organoids, has enabled detailed investigation of RSV–host interactions under physiologically relevant conditions [27]. These models demonstrate that RSV preferentially infects ciliated epithelial cells and induces characteristic epithelial remodeling, including syncytium formation, impaired ciliary function, epithelial damage, goblet cell hyperplasia, and mucus hypersecretion, all of which contribute to impaired mucociliary clearance [74,75,84]. RSV infection of differentiated airway epithelial cultures also triggers robust innate immune activation, including type III interferon responses and the production of inflammatory chemokines such as C-X-C motif chemokine ligand (CXCL)8 and C-C motif chemokine ligand (CCL)5, which contribute to both antiviral defence and immunopathology [74]. These responses are now recognized as major determinants of disease severity, particularly in paediatric lower respiratory tract infections. Beyond enabling mechanistic studies, these models provide quantifiable and physiologically relevant readouts extending beyond viral replication, including epithelial integrity, mucociliary function, and inflammatory signaling. Such parameters enhance the translational relevance of preclinical evaluation of RSV vaccines and monoclonal antibody therapies and may help bridge the gap between conventional in vitro studies and clinical outcomes [27,83].

##### Emerging Respiratory Viruses

Respiratory organoid models have been increasingly applied to the study of emerging and re-emerging respiratory viruses, including human metapneumovirus (HMPV), adenoviruses, rhinoviruses, and highly pathogenic avian influenza viruses such as H5N1 and H7N9 [27,76,77,78,85,86,87]. Their rapid implementation following the emergence of novel pathogens highlights one of the major advantages of organoid technology: the ability to provide physiologically relevant human models when conventional experimental systems are limited [10]. Because respiratory organoids can be generated from different anatomical regions of the airway, they enable comparative analyses of viral tissue tropism, cellular susceptibility, replication kinetics, and virus-induced host responses across distinct epithelial cell populations [63]. These features make respiratory organoids valuable platforms for investigating viral pathogenesis, evaluating antiviral therapeutics, and supporting the preclinical assessment of vaccine candidates. In addition, comparative studies performed in organoid systems may facilitate the identification of conserved viral targets and host factors that could contribute to the development of broadly protective vaccines against emerging respiratory viruses [10,76].

#### 4.1.2. Enteric Viruses

The gastrointestinal tract represents one of the largest mucosal surfaces of the human body and serves as an important site of infection for numerous viral pathogens. Enteric viruses, including rotavirus, norovirus, and enteroviruses, remain major causes of gastrointestinal disease, particularly among infants, young children, elderly individuals, and immunocompromised patients. Their interaction with the intestinal epithelium, mucus barrier, microbiota, and mucosal immune system creates unique challenges for the development of effective oral vaccines and mucosal immunization strategies [8,88,89]. Historically, studies of enteric viruses have been limited by the lack of physiologically relevant human models. Many important pathogens, particularly human noroviruses, could not be efficiently propagated in conventional cell culture systems, restricting investigations of viral replication, tropism, and host responses. The development of human intestinal organoids and enteroids has transformed this field by providing three-dimensional models that reproduce key features of intestinal epithelium, including cellular differentiation, epithelial polarity, barrier function, and innate immune responses [26,35,90]. Intestinal organoids preserve important epithelial antiviral mechanisms, including interferon and cytokine responses, allowing investigation of virus-induced host responses in a human-specific context. Compared with immortalized cell lines, these models enable analysis of cell type-specific susceptibility, viral replication, and tissue responses under conditions that better reflect the in vivo intestinal environment [8,88]. Because oral vaccines must overcome multiple intestinal barriers, including mucus, epithelial integrity, antigen uptake mechanisms, and local immune regulation, intestinal organoids provide valuable platforms for evaluating vaccine candidates and mucosal immune responses. Increasingly complex systems incorporating immune cells or microbiota-derived components may further improve prediction of vaccine efficacy and individual variability in mucosal immunity [89,91].

##### Human Norovirus

Human intestinal enteroids represent a breakthrough in norovirus research because they enabled productive cultivation of human noroviruses after decades of limitations with conventional culture systems. Organoid-based models have demonstrated the importance of host factors such as histo-blood group antigens (HBGAs), bile acids, and epithelial differentiation state in determining norovirus susceptibility (Table 3) [35,92]. These models also reveal donor-dependent differences in infection susceptibility associated with fucosyltransferase 2 (FUT2)-dependent HBGA expression, providing insights into host variability and mechanisms relevant to mucosal vaccine development. Intestinal organoids may support evaluation of antibody-mediated inhibition of viral attachment and replication as well as assessment of vaccine responses against genetically diverse norovirus strains [88].

##### Rotavirus

Human intestinal organoids and enteroids have substantially improved understanding of rotavirus infection by reproducing epithelial differentiation states that influence viral susceptibility. These models demonstrate preferential infection of differentiated enterocytes and allow investigation of host restriction mechanisms controlling viral replication [43,93]. Rotavirus infection induces epithelial injury and activation of innate antiviral pathways, including interferon-associated responses, providing measurable endpoints for studying protective immunity and vaccine-induced epithelial responses [93,94]. Organoid systems therefore complement conventional models used for evaluation of rotavirus biology and vaccine-related immune mechanisms.

##### Enteroviruses

Human intestinal organoid and enteroid systems provide physiologically relevant models for studying enterovirus infections by reproducing intestinal epithelial differentiation and cellular heterogeneity. These models have been used to investigate poliovirus, enterovirus A71 (EV-A71), and coxsackievirus infections, demonstrating virus-specific epithelial tropism, replication kinetics, and antiviral responses [95,96]. Compared with conventional epithelial cultures, intestinal organoids allow investigation of receptor-dependent susceptibility and host–virus interactions within a more representative tissue environment. These approaches contribute to understanding viral fitness, attenuation mechanisms, and host responses relevant to enterovirus vaccine development [95,96].

#### 4.1.3. Hepatotropic Viruses

Hepatotropic viruses, particularly hepatitis B virus (HBV), hepatitis C virus (HCV), and hepatitis E virus (HEV), remain major causes of acute and chronic liver disease worldwide [97]. Their ability to exploit the unique tolerogenic immune environment of the liver promotes viral persistence and immune evasion, presenting major challenges for the development of both prophylactic and therapeutic vaccines [98,99]. Progress in understanding hepatotropic viral infections has long been limited by the lack of experimental models that accurately reproduce human liver physiology. Conventional hepatoma cell lines and animal models have provided valuable insights into viral replication but incompletely reflect human hepatic architecture, metabolic activity, and innate immune responses. Human liver organoids have emerged as physiologically relevant models that preserve key features of liver tissue, including hepatocyte differentiation, metabolic function, and donor-specific characteristics, thereby enabling more faithful investigation of virus–host interactions [10,36,100]. Liver organoids provide versatile platforms for studying viral entry, replication, innate immune activation, and tissue-specific antiviral responses under physiologically relevant conditions [100,101]. Recent advances incorporating non-parenchymal and immune cells have further increased the relevance of these models for investigating chronic liver inflammation and evaluating vaccine-induced immune responses [101,102].

##### Hepatitis B Virus

HBV remains a major cause of chronic viral hepatitis worldwide despite the availability of effective prophylactic vaccines [97]. While current vaccination strategies provide strong protection against new infections, they do not eliminate established chronic HBV infection, highlighting the need for therapeutic approaches capable of restoring antiviral immunity and promoting functional cure. Human liver organoids have provided valuable models for investigating HBV infection under more physiologically relevant conditions than conventional hepatoma cell lines. Unlike transformed hepatocyte models, which may require exogenous expression of sodium taurocholate transporting polypeptide (NTCP) receptor to achieve efficient HBV susceptibility, differentiated human liver organoids preserve hepatocyte-specific characteristics and support HBV entry and replication in a more physiologically relevant hepatic context [103,104]. Organoid-based studies have improved understanding of HBV–host interactions, including viral persistence, hepatocyte responses, and regulation of antiviral signaling pathways (Table 4). These models enable investigation of infection-associated changes in gene expression and host responses within a human hepatic environment, providing insights into mechanisms that contribute to viral persistence and immune evasion [103]. Patient-derived liver organoids represent a further advance by allowing investigation of donor-specific differences in HBV susceptibility, antiviral responses, and disease-associated molecular characteristics. Such personalized platforms may support the evaluation of therapeutic vaccine strategies aimed at enhancing antiviral T-cell responses and achieving functional cure in chronic hepatitis B.

##### Hepatitis C Virus

Although direct-acting antiviral therapies have transformed the management of HCV infection, an effective prophylactic vaccine remains an important unmet need due to continued viral transmission, risk of reinfection, and extensive genetic diversity of circulating viral strains. The development of an HCV vaccine remains challenging because of rapid viral evolution and multiple mechanisms that allow the virus to evade host immune responses. Human liver organoid systems have provided physiologically relevant platforms for investigating HCV infection by preserving important features of hepatic organization, including hepatocyte polarization, three-dimensional architecture, and cellular functions involved in viral entry and replication. Compared with conventional hepatoma cell cultures, organoid-based models allow more detailed analysis of the spatial organization of host factors involved in HCV entry and viral–host interactions. In polarized hepatoma organoids, HCV entry was shown to involve a sequential interaction with cellular receptors and tight junction-associated factors, demonstrating the importance of hepatocyte organization for productive infection [105]. More recent studies using patient-derived and stem cell-derived liver organoids have expanded understanding of HCV persistence and long-term consequences of infection. HCV-infected liver organoids were shown to support persistent low-level viral replication and to reproduce transcriptional changes associated with altered hepatocyte differentiation, impaired interferon signaling, and disease progression, providing insights into mechanisms that may contribute to chronic liver injury [107]. The development of more complex multicellular liver organoid systems has further improved modeling of HCV-associated pathology by incorporating additional hepatic components and enabling investigation of interactions between viral infection, metabolism, and inflammatory responses. Such platforms may become valuable tools for evaluating antiviral strategies, identifying host factors involved in viral persistence, and supporting future vaccine approaches aimed at inducing protective immune responses against HCV [106].

##### Hepatitis E Virus

HEV is an important cause of acute viral hepatitis worldwide and represents a particular clinical concern among pregnant women, elderly individuals, and immunocompromised patients, in whom infection may result in severe or chronic disease [110]. Despite the availability of licensed vaccines in some regions, improved understanding of HEV pathogenesis and host–virus interactions remains important for developing broader preventive strategies. Human liver organoid systems have provided new opportunities to investigate HEV infection in a more physiologically relevant hepatic environment than conventional cell culture models. These systems preserve hepatocyte differentiation and tissue-specific functions, enabling analysis of viral replication, host susceptibility, and cellular responses during infection. Organoid-based approaches have contributed to understanding how hepatic cellular organization influences HEV infection and provide platforms for evaluating antiviral interventions and vaccine-related immune strategies [108,109]. Because HEV differs biologically from HBV and HCV, including its zoonotic transmission potential and distinct replication strategy, human liver organoid models may help identify virus-specific as well as shared mechanisms involved in hepatotropic viral infections. Future integration of immune components into HEV-permissive organoid systems may further improve evaluation of protective immune responses relevant to vaccine development.

#### 4.1.4. Neurotropic Viruses

Neurotropic viruses are a distinct group of pathogens capable of infecting the central nervous system (CNS), causing encephalitis, congenital neurological abnormalities, chronic neurological impairment, or persistent infection. Experimental investigation of CNS-targeting viruses remains challenging because access to human neural tissue is limited and animal models frequently fail to reproduce human-specific features of neural development, receptor expression, and immune responses. Human brain organoids have emerged as powerful models for studying neurotropic viral infections because they reproduce key aspects of human neurodevelopment, including neural progenitor populations, neurons, astrocytes, and region-specific developmental programs. Generated from pluripotent stem cells, cerebral organoids reproduce aspects of fetal human brain organization and provide physiologically relevant systems for studying viral tropism, neurodevelopmental injury, and antiviral responses [18,111]. Unlike conventional neuronal cell cultures, brain organoids preserve cellular heterogeneity and developmental gradients that influence viral susceptibility and neuropathogenesis. Consequently, they have become important platforms for investigating mechanisms of viral entry, cell-type-specific infection, neuroinflammation, and potential vaccine safety assessment, particularly for pathogens associated with congenital or neurological disease [38,112]. From a vaccinology perspective, brain organoids provide opportunities to evaluate neurovirulence of vaccine candidates, characterize attenuation mechanisms, and assess whether vaccine-induced antibodies or immune responses protect neural tissues from infection-associated damage.

##### Zika Virus

The Zika virus epidemic represented a major milestone in the application of brain organoids for infectious disease research. The association between maternal ZIKV infection and congenital microcephaly highlighted the limitations of conventional experimental models, which poorly reproduced human cortical development. Using cerebral organoids, several studies demonstrated that ZIKV preferentially infects neural progenitor cells, resulting in reduced progenitor proliferation, increased apoptosis, premature neuronal differentiation, and impaired cortical development (Table 5) [38,47]. These findings provided direct experimental evidence linking ZIKV infection with developmental abnormalities observed in affected infants. Subsequent studies showed that ZIKV infection induces activation of interferon pathways, inflammatory signaling, oxidative stress responses, and cell death mechanisms [48]. Brain organoids also enabled comparison of viral strains with different neurovirulence profiles and provided platforms for evaluating antiviral compounds and antibody-mediated protection [48,112]. Because congenital ZIKV infection remains a major target for vaccine development, cerebral organoids may contribute to evaluating whether vaccine-induced immune responses prevent infection-associated neural damage.

##### Japanese Encephalitis Virus and West Nile Virus

Japanese encephalitis virus (JEV) and West Nile virus (WNV) are neuroinvasive flaviviruses responsible for severe encephalitis worldwide. Brain organoid studies have demonstrated that flaviviruses can infect neuronal populations and induce cellular responses associated with neuronal injury and inflammation. Although organoid studies for JEV and WNV remain less extensive than those for ZIKV, neural organoid models have provided insights into neuronal susceptibility, viral replication, and inflammatory responses within human neural tissue. These approaches may help define mechanisms associated with neuroprotection and improve evaluation of vaccine-induced immunity, particularly for comparing protective responses elicited by existing JEV vaccines with pathogenic mechanisms observed during infection [38,48].

##### Herpes Simplex Virus

Herpes simplex virus type 1 (HSV-1) establishes lifelong latency in sensory neurons and may cause severe herpes encephalitis following viral reactivation. Human brain organoids have enabled investigation of HSV-1 neuroinvasion and virus-induced neuronal injury within a human developmental context. Studies using cerebral organoids demonstrated neuronal infection accompanied by activation of innate immune pathways, inflammatory responses, and neuronal damage [113,114,117]. These models provide valuable systems for studying host–virus interactions and evaluating strategies aimed at enhancing antiviral immunity against HSV-associated neurological disease.

##### Cytomegalovirus

Human cytomegalovirus (HCMV) is the leading infectious cause of congenital neurological disorders. Because brain organoids reproduce important features of fetal neurodevelopment, they have become valuable models for investigating congenital HCMV infection. Experimental studies have demonstrated that HCMV infection disrupts neural progenitor proliferation, neuronal differentiation, migration, and cortical organization, reproducing several features observed in congenital infection [115,116]. These findings are highly relevant for vaccine development because prevention of maternal infection and congenital transmission represents the primary objective of HCMV vaccine strategies. Brain organoids may therefore provide useful platforms for assessing whether vaccine-induced antibodies or antiviral immune responses prevent infection of developing neural tissue.

### 4.2. Bacterial Infections

#### 4.2.1. Gastrointestinal Pathogens

The gastrointestinal tract is continuously exposed to a diverse community of commensal microorganisms and enteric bacterial pathogens. Species such as *Helicobacter pylori*, *Salmonella enterica*, pathogenic *Escherichia coli*, *Shigella* spp., and *Clostridioides difficile* interact with the intestinal and gastric epithelium through mechanisms involving epithelial adhesion, invasion, toxin production, and modulation of host immune responses. Understanding these host–pathogen interactions are essential for identifying protective antigens and developing effective mucosal vaccines [89,118]. Conventional epithelial cell lines and animal models have substantially advanced the understanding of bacterial pathogenesis but fail to fully reproduce the structural organization, cellular heterogeneity, and innate immune functions of the human gastrointestinal mucosa. Human gastric and intestinal organoids overcome many of these limitations by preserving epithelial polarity, barrier integrity, mucus production, and tissue-specific signaling pathways, providing physiologically relevant models for investigating bacterial infection and host responses [10,26,119]. These models enable detailed investigation of bacterial colonization, epithelial invasion, toxin-mediated injury, and activation of innate immune pathways within an authentic human epithelial environment. Infection of intestinal and gastric organoids induces production of inflammatory mediators, including interleukin (IL)-8, CXCL1, CXCL2, tumour necrosis factor (TNF)-α, and antimicrobial peptides, thereby facilitating characterization of early epithelial responses that contribute to pathogen clearance and mucosal inflammation [11,89]. Moreover, co-culture systems incorporating immune cells and components of the gut microbiota further enhance the physiological relevance of organoid models and enable investigation of mucosal immune responses relevant to vaccine development.

##### *Salmonella enterica* 

*Salmonella enterica* remains one of the leading causes of foodborne bacterial infections worldwide and is widely used as a model for studying intestinal invasion and mucosal immunity. Following infection, *Salmonella* crosses the intestinal epithelium, invades epithelial cells through a type III secretion system (T3SS), and triggers inflammatory responses that contribute to both pathogen clearance and tissue injury [120,121]. Human intestinal organoids have provided physiologically relevant models for investigating these early host–pathogen interactions (Table 6). Microinjection of *Salmonella* into the organoid lumen reproduces the natural apical route of infection and has demonstrated epithelial invasion, disruption of barrier integrity, activation of nuclear factor kappa-light-chain-enhancer of activated B cells (NF-κβ) signaling, and production of pro-inflammatory mediators including IL-8, IL-6, CXCL1, and TNF-α [122,123]. Organoid studies have further shown that intestinal epithelial cells actively coordinate innate antibacterial responses and contribute to epithelial regeneration following bacterial injury, highlighting their role in maintaining mucosal homeostasis [124]. From a vaccinology perspective, intestinal organoids provide valuable platforms for evaluating bacterial virulence factors, host innate immune responses, and live-attenuated vaccine strains under physiologically relevant conditions. These models may facilitate identification of correlates of protective mucosal immunity and support the development of next-generation oral *Salmonella* vaccines [89,125].

##### *Shigella flexneri* 

*Shigella flexneri* is the principal causative agent of bacillary dysentery and causes disease through invasion of the colonic epithelium, intracellular replication, and direct cell-to-cell spread. Human intestinal enteroid and organoid models have provided physiologically relevant systems for investigating these host–pathogen interactions, overcoming limitations of conventional epithelial cell cultures [126,127]. Organoid-based studies have demonstrated that *Shigella* preferentially invades polarized intestinal epithelial cells, resulting in intracellular replication, epithelial injury, disruption of barrier function, and activation of epithelial inflammatory responses [127]. These models have improved understanding of how epithelial organization and polarity influence bacterial invasion and mucosal responses while preserving key features of the human intestinal environment [86]. From a vaccinology perspective, intestinal organoid models represent promising platforms for evaluating attenuated *Shigella* vaccine candidates, bacterial antigens, and mucosal immune responses under physiologically relevant conditions.

##### *Helicobacter pylori* 

Among bacterial pathogens investigated using organoid technology, *Helicobacter pylori* represents one of the best examples of how three-dimensional human tissue models can improve understanding of chronic host–microbe interactions. Colonization of the gastric mucosa involves long-term communication between bacterial virulence factors and gastric epithelial cells, processes that are difficult to reproduce using conventional monolayer cultures. Human gastric organoid systems reproduce key features of gastric gland architecture, epithelial differentiation, and tissue organization, providing physiologically relevant models for studying *H. pylori* colonization and epithelial responses [128,129,131]. Studies using gastric epithelial models have demonstrated that *H. pylori* virulence factors, particularly the cytotoxin-associated gene A (CagA) protein delivered through the bacterial type IV secretion system, activate host signaling pathways including NF-κB, mitogen-activated protein kinase (MAPK), and β-catenin-associated pathways, contributing to inflammatory responses, altered epithelial proliferation, and mechanisms involved in gastric carcinogenesis [132,133]. Gastric organoid-based infection models provide opportunities to investigate how *H. pylori*-induced inflammatory signaling influences epithelial responses, barrier function, and tissue homeostasis under human-specific conditions [131]. In addition, studies using gastric stem cell models have demonstrated that chronic *H. pylori* colonization can influence gastric stem cell activation and epithelial differentiation programs, contributing to persistent tissue remodeling during infection [134]. From a vaccinology perspective, gastric organoids represent valuable platforms for evaluating candidate vaccines against *H. pylori* by enabling analysis of bacterial adhesion, epithelial responses, inflammatory signaling, and preservation of gastric tissue integrity under physiologically relevant conditions [89,129].

##### *Clostridioides difficile* 

*Clostridioides difficile* remains one of the most important causes of healthcare-associated diarrhea and antibiotic-associated colitis worldwide. Disease severity is primarily determined by the activity of two major exotoxins, toxin A (TcdA) and toxin B (TcdB), which disrupt epithelial barrier function through glucosylation of Rho-family GTPases, leading to cytoskeletal rearrangement, loss of tight junction integrity, epithelial injury, and inflammatory activation [135,136]. Human intestinal organoid and enteroid systems have provided physiologically relevant models for investigating toxin-mediated epithelial damage and host responses. Exposure of intestinal organoids to *C. difficile* toxins reproduces key pathological features observed during infection, including epithelial barrier disruption, altered cell viability, and activation of inflammatory signaling pathways [130]. These models demonstrate that intestinal epithelial cells actively respond to toxin exposure by producing inflammatory mediators, including IL-8 and other chemokines, thereby contributing to mucosal inflammation [89,130]. Because current vaccine approaches against *C. difficile* primarily focus on neutralization of TcdA and TcdB rather than prevention of colonization, intestinal organoids represent valuable platforms for evaluating toxoid-based vaccines, monoclonal antibodies, and other immunotherapeutic strategies. By enabling measurement of epithelial protection, barrier preservation, and modulation of inflammatory responses, organoid systems may improve preclinical assessment of next-generation interventions against *C. difficile* infection [130].

#### 4.2.2. Respiratory Pathogens

Respiratory bacterial infections remain a major cause of global morbidity and mortality despite the availability of antimicrobial therapies and vaccines against selected pathogens. Increasing antimicrobial resistance further emphasizes the need for improved vaccines and more predictive experimental models of bacterial pathogenesis. The respiratory epithelium represents an active immunological barrier that protects against inhaled pathogens through mucus production, mucociliary clearance, secretion of antimicrobial molecules, epithelial repair mechanisms, and regulation of inflammatory responses. Conventional models based on immortalized epithelial cell lines and animal systems have provided important insights into bacterial infection biology; however, they often fail to reproduce the cellular diversity, tissue organization, and human-specific responses of the respiratory tract. Human airway and lung organoids have therefore emerged as physiologically relevant platforms for studying respiratory host–pathogen interactions and epithelial responses [27,137]. Depending on their differentiation strategy, respiratory organoid models contain airway basal cells, ciliated epithelial cells, goblet cells, secretory populations, and alveolar type I and type II pneumocyte-like cells. More advanced systems incorporating immune cells, endothelial components, or fibroblasts enable investigation of multicellular interactions involved in inflammation, tissue repair, and host defense mechanisms [137]. Respiratory organoids provide valuable platforms for investigating bacterial adhesion, epithelial barrier disruption, inflammatory signaling, antimicrobial responses, and tissue regeneration following infection. By preserving human-specific epithelial characteristics, these models complement conventional experimental approaches and offer opportunities for evaluating mucosal vaccine candidates, bacterial virulence mechanisms, and correlates of protective immunity. Future integration of vascularized lung organoids, immune-enhanced co-culture systems, lung-on-chip technologies, and single-cell multi-omics approaches is expected to further improve the predictive value of respiratory organoid models. These advances may accelerate preclinical evaluation of respiratory bacterial vaccines and support the development of next-generation mucosal immunization strategies.

##### *Mycobacterium tuberculosis* 

*Mycobacterium tuberculosis* (*M.tb*) remains one of the most important respiratory bacterial pathogens worldwide and a major target for improved vaccine development. Despite widespread BCG vaccination, protection against adult pulmonary tuberculosis remains variable, highlighting the need for vaccines capable of inducing stronger and more durable cellular immunity [138,139]. A major challenge in tuberculosis research is the study of granulomas, complex multicellular structures composed of infected macrophages, lymphocytes, fibroblasts, and extracellular matrix components that both restrict bacterial dissemination and support long-term bacterial persistence. Conventional two-dimensional cultures cannot reproduce this organization, whereas animal models frequently show differences in granuloma formation compared with human disease [140,141,142]. Human lung organoid-based and three-dimensional immune co-culture models have emerged as valuable platforms for investigating *M.tb*–host interactions within a more physiologically relevant environment (Table 7). Early studies using airway organoids demonstrated that mycobacteria interact with respiratory epithelial cells and induce transcriptional changes associated with antimicrobial defence, inflammatory signaling, and epithelial responses [143]. More recent lung organoid infection models have shown that *M.tb* can invade human lung epithelial organoids, persist within epithelial and macrophage-containing systems, and induce host responses involving inflammatory cytokines, chemokine production, and Toll-like receptor (TLR)/NF-κβ-associated pathways [144,145]. Integration of macrophages and immune components into lung organoid systems further improves their ability to reproduce key aspects of tuberculosis pathogenesis, including epithelial–immune cell communication, intracellular bacterial survival, and tissue-level inflammatory responses. Three-dimensional bioengineered models have additionally provided insights into granuloma-associated mechanisms and the role of the extracellular microenvironment during infection [146]. From a vaccinology perspective, human lung organoid and immune-enhanced 3D models provide opportunities to evaluate vaccine-induced cellular responses, macrophage–T-cell interactions, and tissue-specific correlates of protection. These platforms may complement traditional animal models during development of next-generation tuberculosis vaccines and host-directed therapeutic strategies [139,145].

##### Other Respiratory Bacterial Pathogens

Beyond *M.tb*, respiratory organoids are increasingly being applied to investigate other clinically important bacterial pathogens of the respiratory tract. By preserving epithelial architecture, cellular diversity, and tissue-specific innate immune responses, airway and lung organoids provide physiologically relevant models for studying bacterial colonization, epithelial injury, and host–pathogen interactions that cannot be fully reproduced using conventional cell cultures [152,153]. *Streptococcus pneumoniae*, the leading bacterial cause of community-acquired pneumonia, has recently been investigated using human lung organoids derived from embryonic stem cells [148]. These models support pneumococcal colonization, epithelial invasion, and inflammatory responses that are difficult to reproduce in conventional animal models, particularly for poorly invasive serotypes such as ST23F. Human lung organoids therefore represent promising platforms for investigating pneumococcal pathogenesis and may facilitate preclinical evaluation of conserved pneumococcal antigens and multivalent vaccine candidates [147,148]. Respiratory organoid models have also substantially improved investigations of *Pseudomonas aeruginosa*, an opportunistic pathogen responsible for chronic airway infections, particularly in patients with cystic fibrosis. Human airway organoids reproduce bacterial biofilm formation, epithelial barrier disruption, and quorum sensing-dependent virulence while enabling simultaneous analysis of host and bacterial transcriptional responses [150]. More recent studies have further demonstrated that *P. aeruginosa* invades differentiated airway organoids through goblet cells, resulting in epithelial damage and bacterial dissemination across the respiratory barrier [149,151]. These models provide valuable platforms for investigating bacterial virulence mechanisms, host immune responses, and antimicrobial therapies, while also supporting the identification of potential vaccine targets. Applications of lung organoids to infections caused by other respiratory pathogens, including *Klebsiella pneumoniae* and *Acinetobacter baumannii*, remain limited. Nevertheless, these multidrug-resistant bacteria have been recognized as important future targets for organoid-based infection models, which may facilitate investigations of epithelial innate immunity, antimicrobial resistance, and preclinical evaluation of novel therapeutic and vaccine strategies [152,153].

#### 4.2.3. Mucosal Bacterial Pathogens

Mucosal surfaces represent the primary interface between the host and numerous bacterial pathogens. Successful colonization of the respiratory, gastrointestinal, and urogenital mucosa requires coordinated interactions with epithelial cells, including bacterial adhesion, nutrient acquisition, immune evasion, and, in some cases, translocation across epithelial barriers. These early host–pathogen interactions are increasingly recognized as critical determinants of infection outcome and constitute attractive targets for prophylactic vaccination [154,155]. Many mucosal bacterial pathogens exhibit a high degree of host and tissue specificity, limiting the translational value of conventional animal models. Human organoids derived from mucosal tissues provide physiologically relevant platforms that preserve epithelial polarity, cellular diversity, barrier function, and tissue-specific signaling pathways, thereby enabling detailed investigation of bacterial colonization and early epithelial responses under human-specific conditions [10,11,89]. Unlike studies focused on invasive disease, organoid-based models allow investigation of the earliest stages of bacterial infection, including epithelial adherence, barrier disruption, innate immune activation, and mechanisms that promote persistent mucosal colonization. These processes are particularly relevant for pathogens such as *Neisseria gonorrhoeae* and *Neisseria meningitidis*, for which asymptomatic carriage and mucosal persistence play central roles in transmission and disease epidemiology [156,157]. From a vaccinology perspective, mucosal organoids provide opportunities to evaluate bacterial adhesins, outer membrane proteins (OMPs), outer membrane vesicles (OMVs), and other surface-associated antigens involved in colonization. Future integration of organoids with immune cells, resident microbiota, and organ-on-chip technologies is expected to further improve the preclinical evaluation of vaccines designed to prevent mucosal colonization and interrupt pathogen transmission [89,152].

##### *Neisseria gonorrhoeae* 

*Neisseria gonorrhoeae*, the etiological agent of gonorrhea, remains one of the most common bacterial sexually transmitted pathogens worldwide and has become a major public health concern because of rapidly increasing antimicrobial resistance and the absence of a licensed vaccine. The development of effective vaccines has been hampered by extensive antigenic variability, multiple immune evasion mechanisms, and the strict adaptation of *N. gonorrhoeae* to the human genital mucosa, which limits the usefulness of conventional animal models [158,159]. Recent advances in organoid technology have provided new opportunities to establish physiologically relevant models of the human female reproductive tract (Table 8). Human endocervical, ectocervical, endometrial, and fallopian tube organoids preserve epithelial polarity, tissue architecture, hormonal responsiveness, and cellular differentiation, thereby providing experimental systems that more closely resemble native genital tissues than conventional monolayer cultures [160,161,162]. Although the application of organoids to *N. gonorrhoeae* infection remains at an early stage, these models are increasingly recognized as promising platforms for investigating bacterial colonization, epithelial barrier interactions, and mucosal immune responses. Three-dimensional reproductive tract organoids are expected to facilitate studies of bacterial adhesion, epithelial invasion, host inflammatory signaling, and tissue-specific mechanisms of infection that cannot be adequately reproduced using immortalized cell lines. Furthermore, organoid-based systems may provide valuable platforms for investigating the influence of sex hormones and epithelial differentiation on gonococcal pathogenesis [163]. From a vaccinology perspective, reproductive tract organoids offer considerable potential for evaluating vaccine candidates targeting conserved gonococcal antigens, including pili, porin B (PorB), transferrin-binding proteins, and OMVs. Integration of organoids with immune cells and components of the genital microbiota is expected to further improve their utility for assessing mucosal antibody responses, inhibition of bacterial colonization, and early correlates of protective immunity. Although these applications remain largely prospective, organoid technology is expected to become an important component of future preclinical platforms supporting gonococcal vaccine development [89,163].

##### *Neisseria meningitidis* 

*Neisseria meningitidis* is an exclusively human-adapted pathogen that commonly colonizes the nasopharynx asymptomatically, with invasive meningococcal disease occurring after bacterial penetration of the mucosal barrier. Because colonization represents the critical first step preceding bloodstream invasion, understanding meningococcal interactions with human respiratory epithelium is essential for improving vaccine strategies aimed not only at preventing disease but also at reducing carriage and transmission [168]. Human respiratory three-dimensional models, including organotypic airway cultures and emerging airway organoid systems, provide physiologically relevant platforms for studying meningococcal attachment, epithelial responses, and mucosal barrier function. These models preserve important features of the respiratory epithelium, including cellular differentiation, mucus production, and epithelial organization that are absent from conventional monolayer cultures [27]. Studies using human nasopharyngeal tissue models have demonstrated that *N. meningitidis* interacts with epithelial surfaces through bacterial adhesins, including type IV pili and opacity-associated proteins, promoting adhesion, microcolony formation, and modulation of host epithelial responses [169,170]. Experimental human airway models have further shown that mucus production and epithelial barrier properties strongly influence meningococcal colonization and inflammatory responses, highlighting the importance of studying infection within a tissue-like environment [164]. From a vaccinology perspective, respiratory organoid-based systems may provide valuable future platforms for evaluating meningococcal vaccine candidates, including outer membrane vesicle (OMV)-based vaccines and conserved surface antigens, while assessing effects on bacterial attachment, epithelial interaction, and mucosal immunity. Such models may be particularly useful for investigating vaccine-mediated reduction of nasopharyngeal carriage, an important factor influencing meningococcal transmission [165].

##### *Bordetella pertussis* 

*Bordetella pertussis*, the causative agent of pertussis, remains an important respiratory pathogen despite widespread vaccination. Although current acellular pertussis vaccines effectively prevent severe disease, their ability to prevent bacterial colonization and transmission is limited, highlighting the need for improved vaccine strategies capable of inducing stronger mucosal immunity. Human airway three-dimensional models have provided new opportunities to investigate interactions between *B. pertussis* and differentiated respiratory epithelium. These systems reproduce infection-relevant features of the airway mucosa, including ciliated epithelial cells, which represent major targets for pertussis colonization. Studies using human airway tissue models demonstrated that *B. pertussis* and its virulence factors, particularly adenylate cyclase toxin, induce epithelial responses associated with inflammatory signaling, altered epithelial function, and tissue damage. These models further revealed differences in bacterial susceptibility and host responses depending on the anatomical origin and differentiation state of airway epithelial tissues, emphasizing the importance of human-specific models for studying pertussis pathogenesis [166,167]. From a vaccinology perspective, airway organoid-based systems provide valuable opportunities to evaluate candidate antigens, toxin-neutralizing strategies, and vaccine approaches designed to enhance local respiratory immunity. By enabling assessment of epithelial responses, inflammatory mediator production, and preservation of mucociliary function, these models may support the development of next-generation pertussis vaccines with improved capacity to reduce colonization and transmission [27,167].

### 4.3. Parasitic Infections

#### 4.3.1. Intestinal Protozoan Infections

Intestinal protozoan parasites remain important causes of gastrointestinal disease worldwide, particularly affecting young children, malnourished populations, and immunocompromised individuals. Their impact is driven not only by direct epithelial damage but also by complex interactions with host cells involving intracellular replication, developmental stage transitions, metabolic adaptation, and immune evasion strategies. These biological characteristics have historically limited mechanistic studies because many protozoan parasites require highly specialized host environments that cannot be adequately reproduced using conventional epithelial cell lines. Human intestinal organoid-based models have provided new opportunities to investigate protozoan–host interactions under physiologically relevant conditions. Compared with traditional two-dimensional cultures, intestinal organoids reproduce key features of the human intestinal epithelium, including cellular differentiation, epithelial polarity, barrier function, mucus production, and lineage-specific host responses. These characteristics enable investigation of parasite attachment, epithelial invasion, intracellular development, host signaling, and tissue responses within a human-derived experimental system [171,172]. A major advantage of intestinal organoids in protozoan research is their ability to model infections caused by parasites with complex life cycles or strict host requirements. Stem cell-derived intestinal models have been adapted to study several protozoan pathogens, providing platforms for analysing parasite development, epithelial responses, and potential therapeutic or vaccine targets [171,173]. Beyond improving understanding of parasite biology, organoid technologies have important implications for vaccinology. Human intestinal models allow evaluation of epithelial protection, parasite-induced tissue injury, inflammatory responses, and potential mechanisms associated with protective mucosal immunity. Future integration of organoids with immune cells, microbiota components, and organ-on-chip technologies may further improve their application for development and testing of vaccines against intestinal protozoan infections.

##### *Cryptosporidium parvum* 

*Cryptosporidium parvum* is an obligate intracellular apicomplexan parasite and one of the most important causes of severe diarrheal disease, particularly among young children and immunocompromised individuals. Despite its global health impact, vaccine development against cryptosporidiosis remains challenging due to limited understanding of protective immunity and the historical lack of physiologically relevant human experimental models. A major limitation in *Cryptosporidium* research has been the inability of conventional intestinal epithelial cell lines to support sustained parasite development. Human intestinal organoids have overcome this limitation by providing differentiated epithelial environments capable of supporting parasite invasion, intracellular development, and progression through multiple stages of the parasite life cycle. These models represent a major advance because they reproduce key aspects of human intestinal infection that cannot be captured using standard two-dimensional cultures [174,175]. Organoid-based studies have demonstrated that intestinal epithelial cells actively participate in host defense against *Cryptosporidium* rather than functioning solely as passive targets (Table 9). Infection induces epithelial transcriptional responses associated with innate immune activation, interferon signaling, inflammatory mediator production, and regulation of epithelial barrier function. Importantly, organoid systems enable investigation of parasite-induced epithelial damage and regenerative responses within a human-specific tissue context, providing insights into mechanisms controlling infection outcome [174]. From a vaccinology perspective, intestinal organoids provide valuable platforms for evaluating vaccine candidates and protective immune mechanisms against cryptosporidiosis. Because they reproduce the natural site of parasite invasion, organoids allow assessment of whether candidate interventions reduce parasite attachment, intracellular development, and epithelial injury. Future integration of organoids with immune cells, microbiota components, and advanced multi-omics approaches may further improve identification of protective antigens and mucosal correlates of immunity required for effective vaccine development [176,177].

#### 4.3.2. Hepatic Protozoan Infections

Protozoan parasites affecting the liver represent a major challenge for infectious disease research because their pathogenic mechanisms frequently involve complex intracellular stages, tissue-specific host interactions, and developmental transitions that are difficult to reproduce using conventional experimental models [181]. Historically, investigation of hepatic protozoan infections has relied mainly on primary hepatocytes, transformed cell lines, and animal models. However, these approaches often fail to fully reproduce human-specific liver physiology, including hepatocyte differentiation, tissue organization, metabolic activity, and interactions with resident immune populations [182,183]. Human liver organoid technologies have therefore emerged as promising platforms for studying parasite–host interactions within a more physiologically relevant hepatic environment [182,184].

##### *Plasmodium* spp.

Malaria remains one of the most important parasitic diseases worldwide, caused predominantly by *Plasmodium falciparum* and *Plasmodium vivax.* Despite recent advances in malaria vaccination, including the introduction of WHO-recommended vaccines targeting pre-erythrocytic parasite stages, the development of more effective vaccines remains a major priority. The hepatic stage of malaria represents a critical target for prophylactic vaccination because successful immune elimination of infected hepatocytes can prevent progression to symptomatic blood-stage infection [178,179]. Following transmission by infected *Anopheles* mosquitoes, *Plasmodium* sporozoites migrate to the liver and invade hepatocytes, where they undergo intracellular development before releasing merozoites into the bloodstream. Historically, studies of this clinically silent stage have relied on primary hepatocytes, hepatoma cell lines, or animal models, which have important limitations related to loss of hepatic differentiation and species-specific differences in parasite development [185,186]. Human liver organoid technologies have emerged as physiologically relevant models for investigating hepatic biology and host–pathogen interactions (Table 9). These systems reproduce important features of human hepatic tissue, including hepatocyte differentiation, polarization, metabolic activity, and tissue organization, providing experimental platforms that more closely reflect human liver physiology than conventional cultures [182,184]. Advanced three-dimensional human hepatic models have enabled investigation of malaria liver-stage infection, including parasite invasion, intracellular development, and host cellular responses within a human-derived hepatic environment [186,187]. From a vaccinology perspective, human liver organoid-based systems represent promising platforms for investigating mechanisms relevant to pre-erythrocytic malaria vaccine development. They may enable evaluation of antibody-mediated inhibition of sporozoite invasion, analysis of infected hepatocyte responses, and identification of correlates of protective immunity. Such approaches are particularly relevant for vaccines targeting circumsporozoite protein (CSP), including RTS,S/AS01 and R21/Matrix-M, while future integration of immune cells and vascular components may further improve their predictive value for next-generation malaria vaccine development [178].

#### 4.3.3. Neurotropic Protozoan Infections

Neurotropic protozoan parasites represent a major challenge for infectious disease research because infection of the central nervous system is associated with severe neurological complications and persistent tissue infection. Conventional experimental models often fail to reproduce the cellular complexity and human-specific responses of neural tissue. Human cerebral organoids derived from pluripotent stem cells have therefore emerged as valuable platforms for investigating host–parasite interactions within a physiologically relevant neural environment [10,17]. From a vaccinology perspective, neural organoid systems provide emerging platforms for studying immune mechanisms associated with protection against neuroinvasive parasites. Although direct vaccine testing in brain organoids remains limited, these models may support identification of parasite targets, analysis of host responses, and evaluation of strategies aimed at preventing persistent infection and tissue damage.

##### *Toxoplasma gondii* 

*Toxoplasma gondii* is an obligate intracellular apicomplexan parasite capable of establishing persistent infections within the central nervous system. Although infection is frequently asymptomatic in immunocompetent individuals, congenital infection and reactivation in immunocompromised patients may result in severe neurological complications. The ability of *T. gondii* to invade neural tissues and establish long-term intracellular persistence makes it an important model for studying parasite–host interactions in the human brain. Traditional studies of cerebral toxoplasmosis have relied mainly on animal models and neuronal cell cultures, which do not fully reproduce the cellular complexity and human-specific responses of neural tissue. Human brain organoids derived from pluripotent stem cells have therefore emerged as valuable platforms for investigating neurotropic infections within three-dimensional human neural environments (Table 9). These models reproduce key features of neural development and contain diverse neural populations, including neural progenitor cells, neurons, and glial cells, enabling investigation of parasite invasion, cellular tropism, and neuroinflammatory responses [10,111]. Recent organoid-based studies demonstrated that *T. gondii* can infect human cerebral organoids, including neural progenitor populations and differentiated neuronal cells, allowing analysis of parasite replication, host transcriptional responses, and mechanisms regulating persistence within neural tissue [180]. These models provide evidence that neural cells actively participate in host defence through induction of interferon-associated pathways and inflammatory responses, while also revealing the complex balance between parasite control and tissue damage. From a vaccinology perspective, brain organoid models represent emerging platforms for investigating protective mechanisms against neuroinvasive parasites. Although direct evaluation of toxoplasmosis vaccine candidates in cerebral organoids remains limited, these systems may contribute to identification of parasite targets, characterization of tissue-specific immune responses, and assessment of strategies aimed at preventing chronic neural infection.

### 4.4. Fungal Infections

Fungal infections have become an increasingly important global health challenge, particularly among immunocompromised individuals, transplant recipients, and critically ill patients. The growing incidence of invasive mycoses together with the emergence of antifungal-resistant pathogens has prompted the World Health Organization to identify priority fungal pathogens requiring intensified research efforts. Despite their substantial clinical burden, no licensed vaccine against any human fungal pathogen is currently available [188,189]. The development of antifungal vaccines has been hindered by the complex biology of fungi, incomplete understanding of protective immunity, and the limited predictive value of conventional experimental models. Human organoids have therefore emerged as promising platforms for investigating fungal pathogenesis in physiologically relevant human tissues. By preserving epithelial architecture, tissue-specific differentiation, and key features of innate immunity, organoids enable investigation of fungal adhesion, epithelial invasion, host inflammatory responses, and early events of tissue colonization under conditions that more closely resemble human infection than conventional monolayer cultures [10,31]. Although organoid applications in medical mycology remain less advanced than those for viral or bacterial pathogens, recent progress in multicellular organoid and organ-on-chip systems is expanding their utility for studying host–fungal interactions and may accelerate the preclinical evaluation of next-generation antifungal vaccines [32].

#### *Candida albicans* 

*Candida albicans* is one of the best-studied fungal pathogens in advanced human epithelial models and an emerging application of organoid technology. As an opportunistic commensal of the oral, gastrointestinal, and vaginal mucosa, *C. albicans* can cause superficial mucosal infections as well as invasive candidiasis in immunocompromised individuals. Human intestinal organoids provide physiologically relevant models that preserve epithelial architecture, cellular diversity, and barrier function, enabling investigation of host–fungal interactions under conditions that more closely resemble the human intestinal mucosa than conventional monolayer cultures [190,191]. Recent studies using human intestinal organoids demonstrated that epithelial cells actively recognize fungal particles and internalize *C. albicans* through Dectin-1-dependent LC3-associated phagocytosis (LAP), highlighting an unexpected role of the intestinal epithelium in fungal sensing and mucosal homeostasis. Organoid models further showed that differentiated epithelial cells contribute directly to antifungal defence by regulating epithelial barrier function and innate immune responses [191]. From a vaccinology perspective, intestinal organoids represent promising platforms for investigating early fungal colonization and evaluating host responses induced by candidate antifungal vaccines. Because these systems reproduce the natural site of *Candida* colonization, they may facilitate assessment of interventions aimed at preventing epithelial colonization, limiting tissue invasion, and identifying mucosal correlates of protective immunity. However, compared with viral and bacterial pathogens, applications of organoid technology to antifungal vaccine development remain at an early stage and require further integration of immune cells and more complex multicellular organoid platforms [192].

## 5. Experimental Applications of Human Organoids in Vaccine Development

Human organoid systems have progressed beyond a purely conceptual application in vaccinology, with several studies providing direct experimental evidence for their use in evaluating vaccine-induced immune responses, comparing vaccine formulations, investigating mechanisms of immunogenicity, and exploring potential correlates of protection (Table 10). The most advanced examples have employed human tonsil-derived immune organoids, which provide a tissue-based experimental system for investigating coordinated B- and T-cell responses and several features of germinal-center-like immunity [39,193,194,195]. Wagar et al. established a human tonsil organoid model that reproduced key features of adaptive immune responses, including antigen-specific antibody production, plasmablast differentiation, class-switch recombination, somatic hypermutation, and affinity maturation. The model was applied to investigate responses to influenza and rabies vaccines, an adenovirus-based SARS-CoV-2 vaccine candidate, and different adjuvant conditions [39]. This study provided direct experimental evidence that human immune organoids can support controlled investigation of vaccine-induced adaptive immunity and allow comparison of different vaccine-related stimuli. Kastenschmidt et al. subsequently used human tonsil organoids to compare different influenza vaccine antigen formats. Distinct vaccine formats generated different patterns of B- and T-cell activation, cellular differentiation, and antibody responses, demonstrating that organoid systems can be used to investigate how vaccine design influences the quality and magnitude of human immune responses [193]. These findings support the use of immune organoids as comparative experimental platforms for vaccine formulation and antigen evaluation. Human tonsil organoids have also been used to investigate mechanisms underlying vaccine-induced immunity. Pudjohartono et al. examined responses to the adenovirus-vectored ChAdOx1 nCoV-19 vaccine and identified a role for plasmacytoid dendritic cells and pDC-derived IFN-α and IL-6 in regulating humoral and cellular responses, including CD4+ T-cell and T follicular helper-cell responses [194]. This study illustrates the potential of organoids to resolve cellular and molecular mechanisms that contribute to the magnitude and quality of vaccine-induced immunity. More recently, Wagoner et al. used human immune organoids to investigate responses to seven influenza vaccines and applied systems-immunology approaches to identify features associated with variability in vaccine responsiveness. The study identified a Th1-associated signature associated with neutralizing antibody responses and subsequently examined this signature in human influenza vaccination datasets [195]. This work represents an important step toward using organoid-derived measurements to identify biological determinants of vaccine responsiveness and candidate correlates of protection. All these studies demonstrate experimentally supported applications of human immune organoids in vaccine research, including assessment of vaccine-induced B- and T-cell responses, comparison of vaccine formats, investigation of mechanisms regulating immunogenicity, and identification of candidate immune signatures associated with vaccine responsiveness.

### 5.1. Concordance with In Vivo and Clinical Vaccine Responses

An important question for the translational value of human organoid models is whether responses observed ex vivo are consistent with those generated following vaccination in humans. Current evidence suggests that organoids can reproduce selected features of human vaccine responses, but the degree of concordance depends on the organoid model, vaccine platform, and biological endpoint. Importantly, direct comparisons with human vaccination data remain relatively limited. The most informative example is provided by Wagoner et al., who identified a Th1-associated signature in human immune organoids that was associated with neutralizing antibody responses to inactivated influenza vaccines [195]. The biological relevance of this organoid-derived observation was subsequently examined in independent human influenza vaccination datasets, in which higher baseline expression of the corresponding Th1 signature was associated with stronger antibody responses following vaccination. This cross-validation provides evidence that organoid-based experiments can identify immune features that remain relevant in human vaccination settings and illustrates their potential value for hypothesis generation and biomarker discovery. However, the available evidence does not yet demonstrate that organoid-based measurements can reliably predict clinical vaccine efficacy or protection. Studies comparing vaccine formulations in tonsil organoids have demonstrated biologically meaningful differences in B-cell, T-cell, and antibody responses, but whether these differences translate into differences in clinical protection has not been established. Similarly, the ability of tonsil organoids to reproduce multiple features of adaptive immunity does not by itself demonstrate quantitative prediction of vaccine responses in vivo [39,193].

Several biological differences may account for incomplete concordance between organoid and in vivo responses. Organoids do not fully reproduce systemic circulation, lymphatic trafficking, endocrine and metabolic influences, tissue-to-tissue communication, or the temporal dynamics of immune responses. Vaccine administration route represents an additional consideration, particularly when comparing mucosal and parenteral vaccination with an ex vivo lymphoid model [195]. Donor variability and differences in organoid composition and maturation may further influence the reproducibility of observed responses.

**Figure 2:**
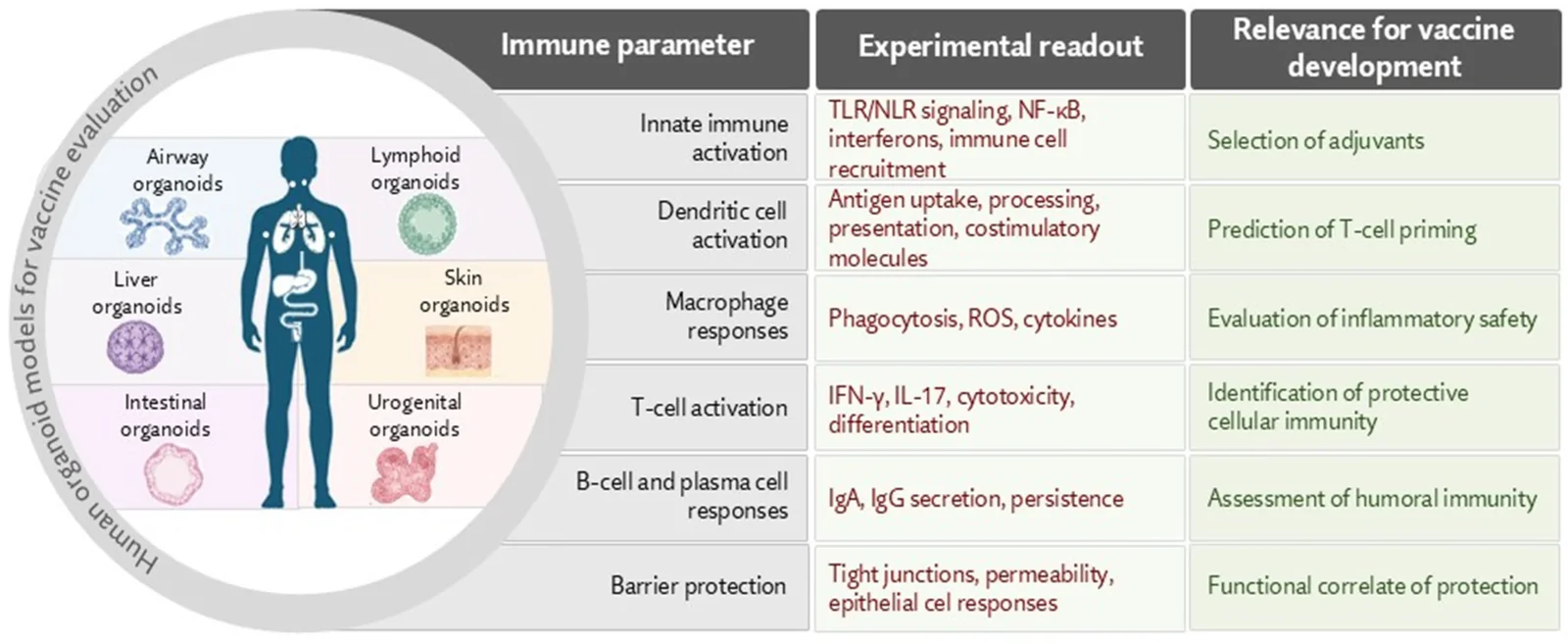
Immune and tissue-protective parameters evaluated using human organoid models during vaccine development. Human organoid systems can be used to investigate multiple components of vaccine-induced responses, including innate immune activation, dendritic-cell and macrophage responses, T-cell activation, B-cell and plasma-cell responses, and preservation of epithelial barrier integrity. Integration of immune cells into organoid models further enables assessment of interactions between tissue-resident cells and adaptive immune components, providing a more physiologically relevant evaluation of vaccine-induced protection.

Therefore, validation should extend beyond demonstrating that an organoid generates an immune response like that observed in humans. More informative approaches require comparison of predefined quantitative or mechanistic endpoints, including neutralizing antibody responses, antigen-specific B- and T-cell responses, transcriptional signatures, cytokine profiles, and candidate correlates of protection (Figure 2). At present, evidence for translational validity is strongest for mechanistic investigation and hypothesis generation, whereas evidence that organoids can directly predict individual vaccine efficacy or clinical protection remains limited.

Human organoids should consequently be regarded as complementary human-relevant models that can bridge mechanistic laboratory studies and subsequent in vivo or clinical investigation. Prospective validation across independent donor cohorts and clinically characterized vaccination studies will be required to determine which organoid-derived parameters have reproducible predictive value.

### 5.2. Patient-Derived Organoids as Emerging Platforms for Personalized Vaccinology

Vaccine responses vary considerably between individuals due to differences in genetic background, age, previous immune exposure, microbiome composition, metabolic status, and underlying diseases. Although current vaccine development strategies have achieved remarkable success, most preclinical evaluation approaches rely on population-average models and do not fully capture the biological diversity that influences vaccine responsiveness. Personalized vaccinology aims to address this limitation by identifying host-specific factors that determine immune activation, durability of protection, and risk of reduced vaccine efficacy.

An important emerging direction in organoid-based vaccinology is the use of patient-derived organoids (PDOs) to investigate inter-individual variability in vaccine responses. Unlike conventional organoid models derived from limited donor sources, PDOs preserve donor-specific genetic and epigenetic characteristics and may reproduce disease-associated alterations in tissue function, inflammatory signaling, and epithelial responses [13,31,200]. Although PDOs are not yet established tools for predicting individual vaccine efficacy, they provide a promising platform for investigating host factors that influence immune activation and tissue responses following vaccination. When combined with autologous immune cells, single-cell sequencing, and multi-omics approaches, patient-specific organoid systems may enable analysis of how genetic and physiological differences affect antigen sensing, cytokine responses, immune-cell recruitment, and establishment of protective immunity. Such approaches may be particularly valuable for studying populations with altered vaccine responsiveness, including elderly individuals, immunocompromised patients, and individuals with chronic inflammatory diseases. Human genetic variation is another important determinant of vaccine responsiveness. Polymorphisms affecting Human Leukocyte Antigens (HLA), pattern-recognition receptors, cytokines, and interferon pathways have been associated with differences in immune responses following vaccination. Because patient-derived organoids retain donor-specific genetic characteristics, they may provide experimental systems for studying how genetic variation influences tissue responses to antigens and adjuvants.

The development of immune-integrated patient-derived organoids represents a further step toward personalized vaccinology. Human immune organoid studies have already demonstrated that complex multicellular systems can reproduce key features of vaccine-induced adaptive immunity, including antigen-specific antibody production, affinity maturation, and T-cell responses [16,39]. Although these studies have primarily used lymphoid organoid systems rather than classical PDOs, they provide an important proof-of-concept for human-specific vaccine testing platforms. Another emerging factor influencing vaccine responsiveness is the interaction between the microbiome and host immunity. Microbial communities regulate epithelial barrier function, innate immune activation, antigen presentation, and systemic immune development. Organoid models combined with defined microbial communities or patient-derived microbiota may provide opportunities to investigate how microbiome composition contributes to variability in vaccine responses. However, microbiome-integrated vaccine organoid systems remain experimental and require further validation before clinical translation. The establishment of organoid biobanks represents an important future direction for precision vaccinology. Collections of organoids derived from individuals with diverse genetic backgrounds, ages, and disease conditions could enable systematic investigation of population variability in vaccine responses. Such resources may support identification of host factors associated with improved immunogenicity or reduced vaccine responsiveness and facilitate development of vaccines optimized for specific populations.

## 6. Challenges and Future Perspectives

**Figure 3:**
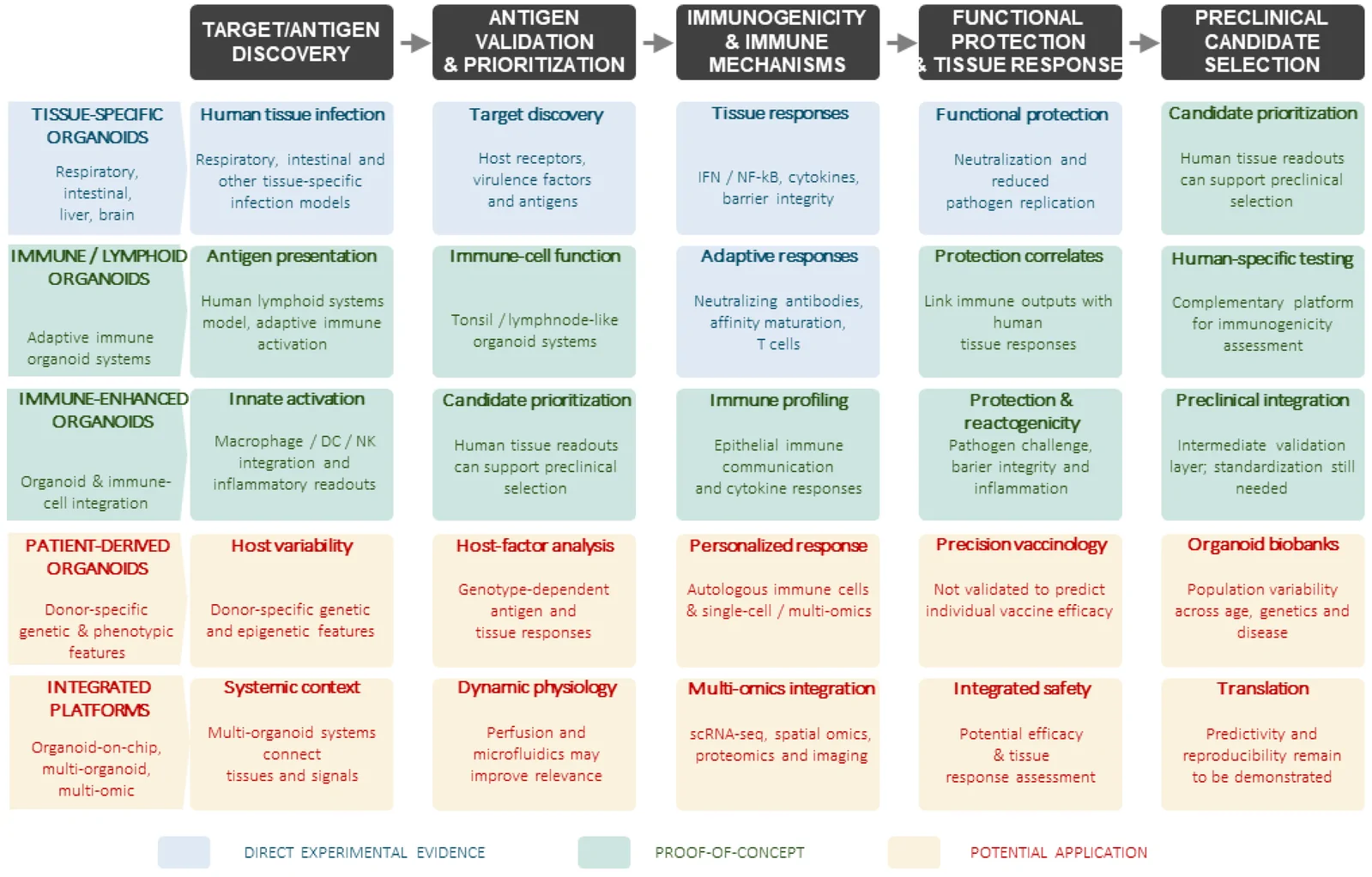
Evidence-based positioning of human organoid platforms across the vaccine-development pipeline. Tissue-specific, immune/lymphoid, immune-enhanced, patient-derived, and integrated organoid platforms contribute to different stages of vaccine development, ranging from antigen discovery and validation to immunogenicity assessment, functional protection, and preclinical candidate prioritization. Applications are classified according to the level of evidence described in the literature reviewed in this article: direct experimental evidence, proof-of-concept, or potential application. Current evidence is strongest for mechanistic studies of human tissue infection, host responses, and selected functional immune and neutralization assays, whereas personalized vaccinology, organoid-on-chip systems, multi-organoid platforms, and computationally integrated approaches remain predominantly at proof-of-concept or prospective stages. Organoids should therefore be considered complementary preclinical platforms rather than replacements for animal models or clinical evaluation. Based on [6,8,12,13,15,16,29,30,32,33,34,39,40,60,64,67,68,69,93,193,196,197,198,199,200].

Despite substantial advances in human organoid technology, several biological, technical, and translational barriers continue to limit its broader implementation in infectious disease research and vaccinology (Figure 3). The major challenges concern model complexity, standardization, scalability, reproducibility, predictive validation, and integration into established preclinical and regulatory frameworks.

One of the principal unresolved challenges is the incomplete cellular and physiological complexity of current organoid systems. Many organoids remain predominantly epithelial and do not fully reproduce the immune, stromal, vascular, neural, or endocrine components of native tissues. Although immune-integrated models can incorporate macrophages, dendritic cells, lymphocytes, and other immune populations, their composition and spatial organization remain difficult to control and can vary between experimental systems [33,39]. Similarly, the absence of functional vascular networks limits long-term maturation, nutrient and oxygen delivery, immune-cell trafficking, and modeling of systemic inflammatory responses. Vascularized organoids, endothelial co-cultures, and organoid-on-chip technologies represent promising approaches, but their integration remains technically challenging [32,34].

Standardization and reproducibility represent another major barrier to translation. Differences in donor characteristics, stem-cell sources, differentiation protocols, extracellular matrix composition, culture conditions, and analytical procedures can generate substantial variation between laboratories [9,34]. The widespread use of poorly defined extracellular matrices such as Matrigel further contributes to batch-to-batch variability and complicates experimental comparison. Future efforts should therefore prioritize chemically defined matrices, standardized differentiation protocols, validated quality-control criteria, and reference organoid systems or biobanks. Establishing agreed criteria for organoid identity, maturity, cellular composition, and functional performance will be important for improving reproducibility and facilitating multicenter validation.

Scalability, cost, and technical complexity also restrict the broader application of organoids, particularly in high-throughput vaccine research. Organoid generation may require specialized expertise, extended culture periods, expensive reagents, and advanced imaging or sequencing platforms. Automation, miniaturization, robotic culture, microfluidics, and standardized high-content imaging may improve throughput and reduce some technical barriers [32,34]. An important challenge will nevertheless be to increase scalability without losing the biological complexity that distinguishes organoids from conventional cell-based systems.

Another major priority is establishing robust validation frameworks. As discussed in Section 5, evidence that organoids reproduce selected human immune responses does not necessarily demonstrate that they can predict clinical vaccine efficacy or protection. Future studies should therefore use predefined endpoints and systematically compare organoid-derived measurements with appropriately matched in vivo and clinical datasets. Standardized benchmarking across independent donors, laboratories, and vaccine platforms will be essential for determining which organoid characteristics have reproducible predictive value.

The integration of organoids with single-cell and spatial multi-omics, advanced imaging, and computational approaches represents an important future direction. These technologies may improve characterization of cellular states and tissue-level responses while enabling identification of multidimensional biomarkers of infection, immunogenicity, and tissue injury. Artificial intelligence and machine-learning approaches may further assist in integrating high-dimensional datasets and identifying complex response patterns. Their successful application, however, will depend on sufficiently large, standardized, and independently validated datasets.

Finally, regulatory acceptance and integration into existing preclinical pipelines remain unresolved. At present, organoids should be considered complementary human-relevant models rather than replacements for animal studies or clinical trials. Their incorporation into regulatory and translational workflows will require evidence of reproducibility, predefined performance criteria, interlaboratory robustness, and a demonstrated relationship between model-derived endpoints and clinically relevant outcomes. Harmonized protocols and validation frameworks will therefore be essential for establishing confidence in organoid-based assays.

Future development should focus not simply on increasing organoid complexity, but on establishing fit-for-purpose models with demonstrable reproducibility and predictive value. Progress will depend on combining appropriate biological complexity with standardized manufacturing, quantitative benchmarking, and systematic validation against human data. These efforts will be essential for determining which organoid platforms can provide meaningful added value to vaccine development and translational infectious disease research.

## 7. Conclusions

Human organoid technology represents a major advance in infectious disease research by providing human-relevant models that bridge the limitations of conventional in vitro systems and animal studies. By preserving key structural and functional features of human tissues, organoids enable investigation of pathogen biology, tissue-specific immune responses, and vaccine-induced protection in environments that better resemble natural infection. The integration of immune components, patient-derived tissues, and advanced molecular approaches expands the potential of organoids for vaccine research by enabling functional evaluation of vaccine targets and investigation of human-specific variability in immune responses. Although challenges related to immune complexity, standardization, scalability, and regulatory validation remain, continued advances in organoid engineering and computational biology are increasing their translational value. Rather than replacing existing preclinical and clinical approaches, organoids are expected to complement current systems by providing mechanistic insights into human immunity that are difficult to obtain using traditional models. Their integration with multi-omics, bioengineering, and computational modelling may ultimately support more predictive approaches for vaccine design and accelerate the development of safer and more effective vaccines.

## Figures and Tables

**Table 1:** Human organoid models and their applications in infectious disease research and vaccine development.

Organoid Type	Main Cellular Components	Representative Pathogens	Applications in Vaccine Development	Key Advantages	References
Respiratory	Basal, ciliated, goblet, club, AT2 cells	SARS-CoV-2, Influenza, RSV	Infection models, neutralization assays, mucosal immunity	Human airway physiology	[27,30]
Intestinal	Enterocytes, Paneth, goblet, enteroendocrine cells	Rotavirus, Norovirus, *Salmonella*, Poliovirus	Oral vaccines, barrier function, antigen uptake	Crypt-villus architecture	[26,35]
Liver	Hepatocytes, cholangiocytes	HBV, HCV, HEV	Vaccine safety, hepatotoxicity, antiviral studies	Human metabolism	[36]
Brain	Neurons, astrocytes, radial glia	Zika, JEV, HSV	Neurovirulence, vaccine safety	Human neurodevelopment	[18,37,38]
Tonsil/Lymph node	B cells, T cells, dendritic cells, stromal cells	Multiple pathogens	Immunogenicity, germinal center reactions	Adaptive immunity	[39]
Multi-organoid systems	Multiple tissues	Systemic infections	Biodistribution, toxicity, immune trafficking	Whole-body interactions	[15]

**Table 2:** Applications of respiratory organoids in studies of viral pathogenesis and vaccine development.

Virus	Organoid Model	Major Biological Findings	Implications for Vaccine Development	References
SARS-CoV-2	Airway, bronchial, alveolar	ACE2/TMPRSS2 distribution, delayed IFN response, epithelial injury	Neutralization assays, variant evaluation, adjuvant screening	[29,63,66,67]
Influenza A/B	Airway, alveolar	Cell tropism, cytokine profile, mucociliary dysfunction	Vaccine strain evaluation, universal vaccine targets	[71,72,73]
RSV	Airway	Syncytium formation, mucus hypersecretion, IFN-λ induction	Evaluation of RSV vaccines and monoclonal antibodies	[27,74,75]
H5N1/H7N9	Airway	Enhanced viral replication, epithelial damage, robust innate immune responses	Pandemic preparedness	[76,77]
Human metapneumovirus	Airway	Ciliated cell infection, innate immune and interferon responses	Vaccine antigen selection	[78]

**Table 3:** Applications of intestinal organoid models in the study of enteric viruses and vaccine development.

Virus	Organoid Model	Main Findings in Organoid Systems	Relevance for Vaccine Development	References
Human Norovirus	Human intestinal enteroids	Productive viral replication, HBGA-dependent susceptibility, bile acid and differentiation effects on infection	Evaluation of antibody-mediated inhibition, antigen responses, strain diversity	[35,92]
Rotavirus	Human intestinal organoids/enteroids	Infection of differentiated enterocytes, host restriction mechanisms, epithelial injury, interferon responses	Assessment of protective immunity and vaccine-induced epithelial responses	[43,93,94]
Enteroviruses (Poliovirus, EV-A71, Coxsackievirus)	Human intestinal enteroids/3D intestinal organoids	Virus-specific tropism, replication kinetics, receptor-dependent susceptibility, antiviral signaling	Investigation of attenuation, replication fitness, and vaccine-related host responses	[95,96]

**Table 4:** Liver organoids as models for hepatotropic virus infection and vaccine development.

Virus	Organoid Model	Major Biological Findings	Implications for Vaccine Development	References
Hepatitis B virus (HBV)	Adult stem cell-derived liver organoids	Physiological NTCP expression, persistent infection, weak innate immune activation	Therapeutic vaccine development, immune restoration, antigen discovery	[103,104]
Hepatitis C virus (HCV)	Hepatocyte organoids	Viral replication, metabolic reprogramming, interferon responses	Conserved antigen identification, antiviral immunity	[105,106,107]
Hepatitis E virus (HEV)	Liver organoids	viral replication, host–virus interactions, and infection-associated cellular responses in a human hepatic environment	Vaccine target validation, antiviral screening	[108,109]

**Table 5:** Applications of brain organoid models in the study of neurotropic viruses and vaccine development.

Virus	Organoid Model	Major Biological Findings	Implications for Vaccine Development	References
Zika virus (ZIKV)	Cerebral organoids, cortical organoids	Infection of neural progenitor cells, reduced proliferation, apoptosis, impaired cortical development, strain-dependent neurovirulence	Evaluation of vaccine-induced protection and prevention of neurodevelopmental damage	[38,47,112]
Japanese encephalitis virus (JEV)	Brain organoids/neural progenitor models	Neuronal infection, neuroinflammatory responses, mechanisms of neuroinvasion	Understanding protective immunity and vaccine-associated neuroprotection	[38]
West Nile virus (WNV)	Neural organoids	Neuronal susceptibility, viral replication, inflammatory responses	Identification of correlates of neuroprotection and evaluation of antiviral strategies	[38]
Herpes simplex virus (HSV-1)	Cerebral organoids	Neuronal infection, innate immune activation, inflammatory responses, neurotoxicity	Investigation of therapeutic vaccine strategies and antiviral immunity	[113,114]
Human cytomegalovirus (HCMV)	Cerebral organoids, cortical organoids	Neural progenitor infection, impaired neurodevelopment, disrupted cortical organization	Evaluation of vaccines preventing congenital infection and fetal brain damage	[115,116]

**Table 6:** Applications of gastrointestinal organoids in studies of bacterial pathogenesis and vaccine development.

Pathogen	Organoid Model	Major Biological Findings	Implications for Vaccine Development	References
*Salmonella enterica*	Intestinal organoids	T3SS-mediated invasion, epithelial barrier disruption, NF-κB activation, stem cell regeneration	Live-attenuated vaccines, mucosal immunity, antigen discovery	[122,123,124]
*Shigella flexneri*	Intestinal organoids	Basolateral invasion, inflammasome activation, pyroptosis, IL-18 secretion	Evaluation of invasion-deficient vaccines, mucosal adjuvants	[126,127]
*Helicobacter pylori*	Gastric organoids	CagA translocation, chronic inflammation, stem cell activation, epithelial transformation	Adhesin- and virulence factor-based vaccines, prevention of chronic colonization	[128,129]
*Clostridioides difficile*	Intestinal organoids	Toxin-mediated epithelial injury, disruption of tight junctions, inflammatory cytokine release	Toxoid vaccines, neutralizing antibodies, barrier protection	[130]

**Table 7:** Respiratory organoid models for bacterial pathogens and their applications in vaccine research.

Bacterial Pathogen	Organoid Model	Major Biological Findings	Relevance for Vaccine Development	References
*Mycobacterium tuberculosis*	Lung organoids; immune-enhanced 3D lung/granuloma models	Granuloma-like organization, epithelial–macrophage crosstalk, cytokine production, intracellular bacterial persistence	Development of TB vaccines, identification of cellular correlates of protection, therapeutic vaccination	[143,144,146]
*Streptococcus pneumoniae*	Lung organoids	Epithelial colonization, bacterial invasion, inflammatory responses	Evaluation of pneumococcal antigens and multivalent vaccine candidates	[147,148]
*Pseudomonas aeruginosa*	Airway organoids	Biofilm formation, epithelial barrier disruption, quorum sensing, host inflammatory response	Identification of virulence-associated vaccine targets and evaluation of host responses	[149,150,151]

**Table 8:** Human organoids used to study mucosal bacterial pathogens and vaccine development.

Pathogen	Organoid Model	Major Biological Findings	Vaccine Implications	References
*Neisseria gonorrhoeae*	Endocervical, ectocervical, endometrial and fallopian tube organoids	Mucosal colonization; epithelial barrier interactions; reproductive tract host–pathogen interactions	Evaluation of conserved vaccine antigens (PorB, pili, OMVs); assessment of mucosal immunity; preclinical vaccine screening	[160,161,163]
*Neisseria meningitidis*	Human airway organoid platforms (emerging application); organotypic nasopharyngeal epithelial models	Nasopharyngeal colonization; epithelial adhesion; mucus and barrier interactions; respiratory tissue models	Evaluation of OMV vaccines, conserved surface antigens, mucosal immunity, reduction of bacterial carriage	[164,165]
*Bordetella pertussis*	Human airway 3D epithelial models/airway organoid platforms	Interaction with ciliated epithelial cells; adenylate cyclase toxin effects; epithelial inflammatory responses; mucociliary dysfunction	Evaluation of mucosal pertussis vaccine strategies; assessment of local epithelial immune response	[166,167]

**Table 9:** Organoid-based models for protozoan infections and their applications in vaccine development.

Protozoan Pathogen	Organoid/3D Model	Major Biological Findings	Vaccine-Related Applications	References
*Cryptosporidium parvum*	Human intestinal organoids	Parasite invasion, intracellular development, epithelial responses, barrier disruption	Evaluation of vaccine targets and mucosal immune protection	[174,175,176]
*Plasmodium falciparum*/ *P. vivax*	Human liver organoids and hepatic 3D models	Sporozoite invasion, liver-stage development, host hepatocyte responses	Evaluation of pre-erythrocytic vaccines and protective immunity	[178,179]
*Toxoplasma gondii*	Human cerebral organoids	Neural infection, parasite persistence, neuroinflammatory responses	Identification of immune mechanisms and vaccine targets	[180]

**Table 10:** Applications of human organoids in vaccine development: established applications, proof-of-concept studies, and prospective uses.

Stage of Vaccine Development	Organoid-Based Application	Technologies/Readouts	Representative Examples/Outcomes	Evidence Status	References
Vaccine candidate evaluation	Evaluation and comparison of human immune responses to vaccine antigens and vaccine formulations in human tonsil organoids	Flow cytometry, ELISA, cytokine profiling, antibody profiling, scRNA-seq	Influenza vaccines; rabies vaccine; adenovirus-based SARS-CoV-2 vaccine	Established application	[39,193]
Immune response characterization	Characterization of antigen-specific B- and T-cell responses and germinal-center-related processes following vaccine stimulation	Flow cytometry, scRNA-seq, antibody assays, immune-repertoire analysis, neutralization assays	Antigen-specific antibody production, B-cell differentiation, plasmablast formation, class-switch recombination, somatic hypermutation and affinity maturation	Established application	[39,193]
Adjuvant and formulation assessment	Evaluation of how vaccine formulation or adjuvant selection influences the magnitude and quality of human immune responses	Cytokine profiling, flow cytometry, antibody assays, transcriptomics	Effects of different influenza vaccine formats and adjuvants on B- and T-cell responses	Proof-of-concept study	[39,193]
Mechanistic characterization of vaccine responses	Identification of cellular and molecular pathways regulating vaccine-induced immune responses	Cell depletion/manipulation, cytokine profiling, scRNA-seq, functional assays	Role of plasmacytoid dendritic cells, IFN-α and IL-6 in ChAdOx1-induced responses	Proof-of-concept study	[194]
Candidate prioritization/correlates of protection	Identification of human immune signatures associated with stronger vaccine responses and potential correlates of protection	Systems immunology, multi-omics, scRNA-seq, predictive modelling	Host- and antigen-associated signatures linked to neutralizing antibody responses to different influenza vaccines	Proof-of-concept study	[195]
Antigen discovery and rational vaccine design	Integration of organoid-derived human immune-response data with antigen discovery, reverse vaccinology and computational vaccine design	Multi-omics, bioinformatics, reverse vaccinology, machine learning, computational modelling	Potential refinement and prioritization of vaccine antigens using human-relevant experimental data	Prospective/potential application	[10,31,32,196,197,198,199]
Preclinical evaluation	Use of human organoids as complementary human-relevant models for assessing vaccine-induced responses and, potentially, protection and tissue-specific effects	Challenge models, imaging, cytokine assays, transcriptomics, proteomics	Potential assessment of vaccine-induced protection and tissue-specific effects before or alongside animal studies	Prospective/potential application	[10,31,32,198,199]

## Data Availability

The original contributions presented in this study are included in the article. Further inquiries can be directed to the corresponding author.

## Abbreviations

ACE2	angiotensin-converting enzyme 2
ALI	air-liquid interface
ASCs	adult stem cells
AT2	alveolar type II cells
BMP	bone morphogenetic protein
CagA	cytotoxin-associated gene A
CCL	C-C Motif Chemokine Ligand
CNS	central nervous system
COVID-19	Coronavirus disease 19
CRISPR-Cas9	Clustered Regularly Interspaced Short Palindromic Repeats (CRISPR) and CRISPR-associated (Cas)
CSP	circumsporozoite protein
CXCL	C-X-C motif chemokine ligand
EGF	epidermal growth factor
ESCs	embryonic stem cells
FGF	fibroblast growth factor
FUT2	fucosyltransferase 2
HBGAs	histo-blood group antigens
HBV	hepatitis B virus
HCMV	human cytomegalovirus
HCV	hepatitis C virus
HEV	hepatitis E virus
HLA	human leukocyte antigens
HMPV	metapneumovirus
HSV	Herpes simplex virus
IFN	interferon
Ig	immunoglobulin
IL	interleukin
iPSCs	induced pluripotent stem cells
JEV	Japanese encephalitis virus
LAP	LC3-associated phagocytosis
LC3	Microtubule-associated protein 1A/1B-light chain 3
MAPK	mitogen-activated protein kinase
*M.tb*	*Mycobacterium tuberculosis*
NF-κβ	nuclear factor kappa-light-chain-enhancer of activated B cells
NLR	nucleotide-binding oligomerization domain-like receptor
NTCP	sodium taurocholate contransporting polypeptide
OMPs	outer membrane proteins
OMVs	outer membrane vesicles
PDOs	patient-derived organoids
PorB	Porin B
PSCs	pluripotent stem cells
RNA-seq	RNA sequencing
RSPO	Roof plate-specific spondin
RSV	respiratory syncytial virus
SARS-CoV-2	severe acute respiratory syndrome coronavirus 2
scRNA-seq	single-cell RNA sequencing
TLR	Toll-like receptor
TMPRSS2	transmembrane serine protease 2
TNF	tumor necrosis factor
T3SS	type III secretion system
Wnt	Wingless-related integration site
WNV	West Nile virus
ZIKV	Zika virus
2D	two-dimensional
3D	three-dimensional
